# Schlieren texture and topography induced confinement in an organic exciton-polariton laser

**DOI:** 10.1038/s41467-025-55875-1

**Published:** 2025-01-18

**Authors:** Florian Le Roux, Andreas Mischok, Francisco Tenopala-Carmona, Malte C. Gather

**Affiliations:** 1 Humboldt Centre for Nano- and Biophotonics, Institute for Light and Matter, Department of Chemistry and Biochemistry, University of Cologne, Köln, Germany; 2 Organic Semiconductor Centre, SUPA, School of Physics and Astronomy, University of St Andrews, St Andrews, UK

**Keywords:** Lasers, LEDs and light sources, Nanoscale devices

## Abstract

Non-linearities in organic exciton-polariton microcavities represent an attractive platform for quantum devices. However, progress in this area hinges on the development of material platforms for high-performance polariton lasing, scalable and sustainable fabrication, and ultimately strategies for electrical pumping. Here, we show how introducing Schlieren texturing and a rough intra-cavity topography in a liquid crystalline conjugated polymer enables strong in-plane confinement of polaritons and drastic enhancement of the lasing properties. In high-*Q* distributed Bragg reflector microcavities, polariton lasing was observed at unprecedented thresholds of 136 fJ per pulse. Morphology tuning also permitted polariton lasing in more lossy metallic microcavities while maintaining a competitive lasing threshold. The facile fabrication of these cavities will drastically reduce the complexity of integrating polariton lasers with other structures and the high conductivity of metallic mirrors may provide a route to electrical pumping.

## Abstract (web summary)

Organic exciton-polaritons represent an attractive platform for quantum devices. Here, authors show how introducing Schlieren texturing and a rough intra-cavity topography in a liquid crystalline polymer enables strong in-plane confinement of polaritons and drastically improved lasing.

## Introduction

Frenkel excitons in organic semiconductors have large binding energies and oscillator strengths^1,2^. When hybridized with the photonic mode of a planar microcavity, they form exciton-polaritons that are stable at room temperature and that show a large Rabi splitting, ${{\hslash }}\Omega$, between the resulting lower polariton (LP) and upper polariton (UP) branches; values ≥ 1 eV have been demonstrated in organic microcavities^3–6^. Nonlinearities are manifest in these systems during polariton lasing, that is when polaritons scatter to macroscopically occupy the ground state and then decay through emission of coherent photons. Polariton lasing has been observed in a wide range of organic microcavities containing single-crystals of anthracene^7^, small organic molecules^8^, polymers^9^, or proteins^10^; where LP lifetimes have been sufficiently long for efficient thermalization of the LP ground state population, this also enabled out-of-equilibrium Bose-Einstein Condensation (BEC).

Reducing the threshold of polariton lasing and BEC is of great importance for fundamental studies and for fascinating applications of these phenomena, e.g., superfluidity of light^11^, optical logic at room temperature^12^, and single-photon detection^13^, because threshold is often directly linked to device performance, e.g., in terms of power consumption and device stability. High photoluminescence quantum yield (PLQY)^14^ and fast exciton decay rates^15^ have proven advantageous in this context. Phonon-mediated polariton relaxation^16^ can also yield a reduction in threshold^17^. More recently, the strategy of aligning the exciton transition dipole moment^18,19^ has been explored for polariton lasers. By using an active layer of a liquid-crystalline conjugated polymer (LCCP) that is macroscopically aligned into its nematic phase, thresholds down to 2.23 pJ of incident pulse energy have been demonstrated^19^, thus putting organic polariton lasers on par with the best organic photon lasers based on vertical-cavity surface-emitting designs^20,21^.

Laterally confining the electric field in planar microcavities via free-stranding microstructures and using lithography to create energy potentials that act as polariton traps has been explored as a strategy to modify the local photonic environment and thus to improve the performance and enrich the characteristics of polariton lasers. However, the resulting devices remain prone to fabrication imperfections; specifically for organic polariton lasers the harsh nature of the processes involved in nanofabrication can cause material degradation which ultimately limits the overall benefits of this approach. Random lasers^22,23^ rely on multiple random scattering events inside a gain medium to achieve optical feedback and population inversion. It has been shown that placing scatterers in planar microcavities can reduce the number of lasing modes available to the system and thus provide a pathway to increasing laser efficiency^24^. This additional level of control over the light path within the cavity might therefore provide an alternative strategy to also achieve lateral confinement in polariton lasers.

In recent decades, the polymorphic nature of liquid crystals has garnered significant scientific interest^25–27^, leading to extensive endeavors to classify the numerous phases and textures and their corresponding ordering. Nematic liquid crystals have the ability to align their director, that is the average local direction of their long molecular axis, parallel to a substrate, and when this alignment is not homogeneous, a Schlieren texture^27,28^ with locally well-defined director orientation can emerge (μm to sub-μm domains). This texture has been recorded in LCCPs^29,30^ and can be regarded as an optical medium in which a high degree of disorder arises from an ensemble of highly ordered domains similar in size to the wavelength of visible light, therefore making it a favorable environment for multiple scattering events at the microscopic scale.

Here, we introduce Schlieren textures with characteristic domain sizes <10 µm in films of the LCCP poly(9,9-dioctylfluorene) (PFO) using a scalable bottom-up approach. Using a subtle interplay between the local dipole orientation and the intra-cavity topography originating from these Schlieren textures, we achieve in-plane confinement of polaritons. We find that for films of 15% β*-*phase PFO, discontinuities in refractive index at domain boundaries together with an average roughness for the active layer of *R*_a_ = 2.6 nm lead to a drastic improvement in polariton laser performance. This performance increase allows us to replace the electrically insulating distributed Bragg reflectors (DBRs) that are conventionally used to form the microcavity by highly conductive metallic mirrors. Metallic mirrors were previously unsuitable for polariton lasers due to their lower reflectivity (e.g., ~95% reflectivity for silver compared with >99.9% for DBRs). The deposition of metallic mirrors via thermal evaporation is substantially simpler, gentler, and more rapid (tens of minutes) than the fabrication of DBRs. In addition, metallic mirrors also offer a direct pathway for charge injection in future electrically pumped polariton lasers.

We first fabricate a high-Q polariton laser made of DBRs sandwiching a film of Schlieren textured PFO. Comparing the spatial pattern of emission under polarized excitation to corresponding polarized transmission micrographs reveals that the emission originates from micro-domains with exciton transition dipole moments closely aligned with the pump polarization. For a given polarization, we observe small high-intensity spots, which undergo a super-linear increase in intensity as the excitation energy increases. Selective excitation of these emission centers facilitates polariton lasing with a threshold down to ${P}_{{{{\rm{th}}}},{{{\rm{DBR}}}}/{{{\rm{DBR}}}}}=136\,{{{\rm{fJ}}}}$ per pulse. This threshold represents a record for both organic polariton lasers and organic vertical surface-emitting photon lasers; it is more than 16 times lower than the best value demonstrated for a cavity containing a macroscopically aligned layer of 15% β*-*phase PFO^19^ and almost two orders of magnitude lower than for a corresponding isotropic polariton laser. We observe an average sub-threshold LP linewidth of ~500 μeV, indicating a long polariton lifetime of *τ*_LP_ > 1 ps and a one-order of-magnitude increase in *Q*-factor over non-textured cavities^19^ and comparable zero-dimensional cavities fabricated using Gaussian-shaped defects^31^. We then elucidate the specific role of the rough topography of the cavity and utilize the improvement in threshold afforded by the Schlieren texture to realize anisotropic polariton lasing in a hybrid metal/DBR cavity with a threshold of ${P}_{{{{\rm{th}}}},{{{\rm{Ag}}}}/{{{\rm{DBR}}}}}=2.67\,{{{\rm{pJ}}}}$ per pulse, as well as in a metal/metal cavity with ${P}_{{{{\rm{th}}}},{{{\rm{Ag}}}}/{{{\rm{Ag}}}}}=15.19\,{{{\rm{pJ}}}}$ per pulse. Despite the metal-induced losses, the performance of the purely metallic polariton laser is on par with current state-of-the-art DBR-based polariton lasers. Careful analysis of the hallmarks of polariton lasing—in particular linewidth and blue-shift—for the different types of mirrors reveals a subtle interplay between lasing threshold and surface roughness.

## Results

### Structure of polariton lasers

**Fig. 1:**
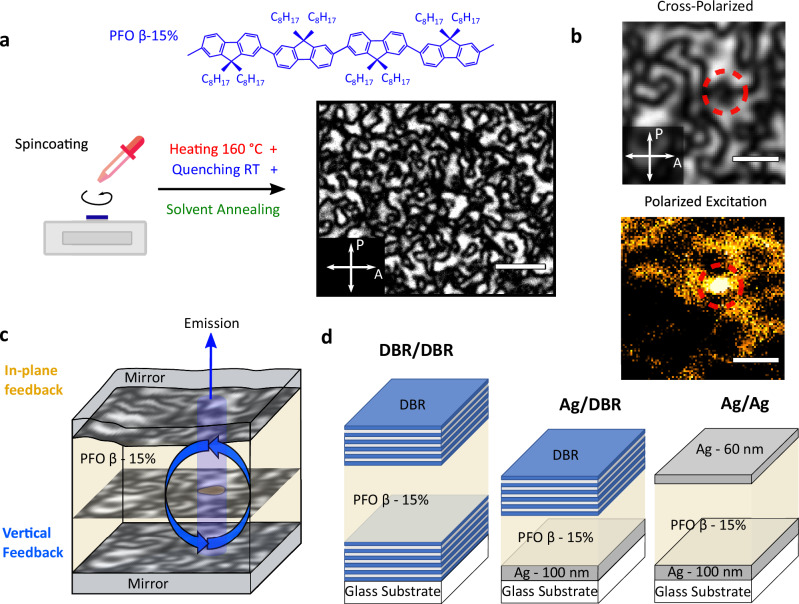
Fabrication and working principle of the Schlieren-textured polariton lasers. **a** Illustration of protocol for forming Schlieren textured active layers of PFO with 15% β-phase content. Chemical structure of the β-phase chain conformation of PFO shown in blue. Polarized transmission optical micrograph of the resulting film placed between a crossed polarizer (P) and analyzer (A) pair. No light is transmitted when the exciton transition dipole moment lies perpendicular to either the polarizer or analyzer, while maximum transmission occurs when the transition dipole moment lies at 45° relative to both the analyzer and polarizer. **b** Polarized optical micrograph (top) and corresponding emission under non-resonant polarized excitation (false color, bottom) on the DBR/DBR cavity surface. The dashed red circle indicates an area of confined emission. **c** Proposed working principle of the Schlieren textured polariton laser: Rough intra-cavity topography combined with local orientation gives rise to in-plane confinement with the cavity mirrors providing vertical feedback. **d** Schematic structure of the dielectric DBR/DBR, hybrid Ag/DBR, and metallic Ag/Ag cavities used in this study. Scale bar in (**a**), 10 μm; in (**b**), 4 μm.

Fig. 1a illustrates our protocol for forming Schlieren-textured active layers of nematic phase PFO through heating (160 °C for 10 min) and rapid quenching to room temperature, and finally, solvent (toluene) vapor annealing for 24 h to induce 15% of the PFO β*-*phase. Also shown is a typical polarized optical micrograph of the resulting film. Fig. 1b shows the characteristic emission pattern at the surface of a microcavity sample and Fig. 1c illustrates the proposed emission mechanism in which in-plane feedback provided by both the Schlieren texture domains and the rough intra-cavity topography couples with the vertical feedback provided by the cavity mirrors.

We fabricated and compared three types of cavities, namely DBR/DBR, Ag/DBR, and Ag/Ag (Fig. 1d, for details on fabrication see Methods). Since coherent emission is expected to arise from aligned micro-domains in the cavities and with its polarization along the direction of alignment, the optical thickness of each cavity design was optimized for these conditions using transfer matrix calculations (TMCs, Supplementary Fig. 9). The LP energy at normal incidence (*θ* = 0°) was tuned to match the (0–1) vibronic peak of the emission of β*-*phase PFO, which is at 2.67 eV (Supplementary Fig. 1); this strategy has proven beneficial for polariton laser perfomance^17,19^.

### In-plane confinement of polaritons under polarized excitation

**Fig. 2:**
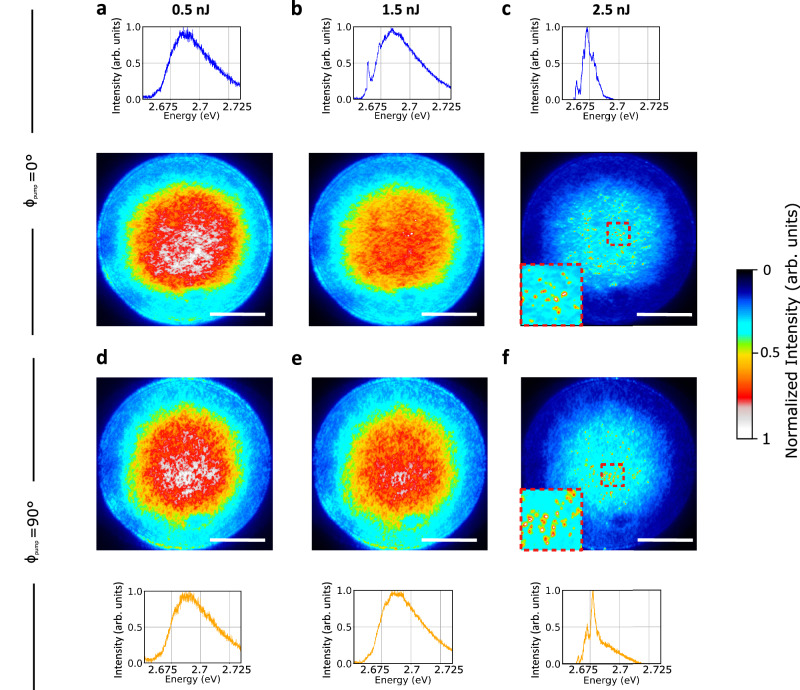
Spatially and spectrally resolved emission from the Schlieren textured DBR/DBR cavity. Spatially and spectrally resolved emission from a DBR/DBR cavity with Schlieren textured active layer upon non-resonant excitation with increasing pulse energy and with orthogonal polarizations, ${\phi }_{{{{\rm{pump}}}}}=0^\circ$ (**a**, **b**, **c**) and ${\phi }_{{{{\rm{pump}}}}}=90^\circ$ (**d**, **e**, **f**). Excitation pulse energies were 0.5 nJ (**a** and **d**), 1.5 nJ (**b** and **e**), and 2.5 nJ (**c** and **f**). Note that non-uniform emission is already present at 0.5 nJ. Additionally, highly localized high-intensity emission spots occur from excitation energies of 1.5 nJ and they are accompanied by clear narrow emission peaks in the spectrum. The insets in (**c** and **f**) show magnifications of the areas marked by red-dashed rectangles in the corresponding main panels. (See Supplementary Information Figs. 2 and  3 for data spanning a wider range of excitation energies.) Scale bars, 50 μm.

Fig. 2 shows the spectrally and spatially resolved emission from a thickness-optimized DBR/DBR cavity under non-resonant pulsed excitation with an extended pump spot (diameter *d*_pump_ ~ 150 μm; wavelength 355 nm; photon energy 3.49 eV; pulse duration 25 ps; repetition rate 250 Hz). The polarization of the pump spot was set to either vertical (${\phi }_{{{{\rm{pump}}}}}=0^\circ$; Fig. 2a–c) or horizontal (${\phi }_{{{{\rm{pump}}}}}=90^\circ$; Fig. 2d–f) and the pulse energy was varied between 500 pJ and 2.5 nJ. Irrespective of pulse energy, we found the spatially resolved emission to be complementary for the two orthogonal polarizations, i.e., areas that appeared bright for ${\phi }_{{{{\rm{pump}}}}}=0^\circ$ tended to be dark for ${\phi }_{{{{\rm{pump}}}}}=90^\circ$. We attribute this to the fact that parallel or close to parallel alignment of the local transition dipole moment ${{{\boldsymbol{\mu }}}}$ with pump polarization ${{{\bf{E}}}}$ leads to a larger dot product ${{{\boldsymbol{\mu }}}}\cdot {{{\bf{E}}}}$ and thus stronger absorption, resulting in brighter emission.

At a pump pulse energy of 500 pJ (2.84 μJ cm^−2^, Fig. 2a, d), the spectra for orthogonal pump polarizations were similar and resembled the LP emission spectra previously observed for an aligned PFO 15% β-phase cavity operating in the sub-threshold regime^19^. Increasing the excitation pulse energy to 1.5 nJ (8.52 μJ cm^−2^) led to the formation of high-intensity, localized emission spots, accompanied by high-intensity narrow emission peaks on the red edge of the spectrum (Fig. 2b, e). These peaks were confined to the region of LP emission; this behavior differs from photonic random lasing where emission is typically observed across the entire fluorophore emission spectrum. At an excitation pulse energy of 2.5 nJ (14.2 μJ cm^−2^), numerous bright spots appeared, and the corresponding spectra were dominated by individual narrow peaks (Fig. 2c, f).

The experimental observations indicate the presence of significant in-plane confinement of polaritons, which is further amplified when the system is operated above threshold. The in-plane confinement of polaritons was further corroborated by finite-difference time-domain (FDTD) simulations (Supplementary Note, Supplementary Fig. 4 and 5), which showed the confinement of the electric field in a circular region of diameter *d* ~ 3 µm. The calculated field overlaps with one of the aligned micro-domains with its amplitude decreasing sharply outside of the domain’s borders, similarly to what was observed in the experiment.

### The role of intra-cavity topography

**Fig. 3:**
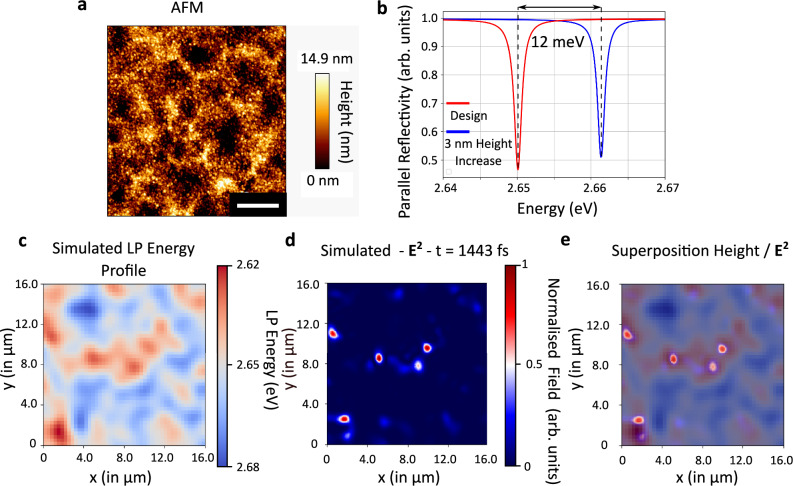
The role of intra-cavity topography. **a** AFM measurement performed on the surface of the DBR/DBR Schlieren cavity showing an average roughness *R*_a_ = 2.6 nm. Scale bar, 2 μm. **b** TMCs of the reflectivity of DBR/DBR cavities with aligned 15% β-phase PFO at the design thickness for a resonance at 2.65 eV (red line) and assuming a 3 nm thickness increase over the design thickness (blue line). The calculation was performed for light polarized parallel to the alignment of the PFO transition dipole moment. **c** Calculated LP energy profile for a randomly generated film topography with a correlation length and amplitude matching the Schlieren cavity measured in (**a**). **d** FDTD simulation of the normalized squared electric field 1443 fs after excitation for the LP energy profile shown in (**c**). **e** Superposition of data from (**c** and **d**) illustrating how the electric field is confined to regions of lower LP energy, i.e., to thicker parts of the film.

Fig. 3a shows an atomic force microscopy (AFM) measurement performed on the DBR/DBR cavity containing a Schlieren textured active layer of PFO with 15% β-phase content. The resulting average roughness of *R*_*a*_ = 2.6 nm is approximately 3 times larger than for a DBR/DBR reference cavity with an active layer of macroscopically aligned 15% β-phase PFO (*R*_*a*_ = 0.88 nm; see Supplementary Fig. 6). Fig. 3b shows reflectance spectra predicted by TMC for two such reference cavities with slightly different thicknesses of the macroscopically aligned PFO layer, demonstrating that a difference in thickness of 3 nm between two adjacent locations across the cavity would lead to a 12 meV shift in LP energy. This local variation in LP energy can reach several tens of meVs between the peaks and troughs in the topography of a cavity containing a Schlieren textured PFO film, which should be sufficient to confine polaritons in the presence of ambient thermal energy.

We next performed FDTD simulations of polariton propagation in the energy landscape defined by the thickness fluctuations of the Schlieren textured PFO film, using randomly generated roughness profiles with different correlation lengths and amplitudes. Fig. 3c–e shows the results for a topography that resembles the topography obtained via AFM. The results from these simulations indicate that the thickness fluctuations alone would be sufficient to confine polaritons, even in the absence of refractive index anisotropies from micro-domain alignment (also see corroborating experimental data, Supplementary Note 4 and Supplementary Fig. 6). Taken together with the polarization-resolved experimental data above, however, we conclude that both the micro-domain alignment and the intra-cavity topography contribute to confining the polaritons in-plane in the Schlieren cavity.

### Mode selection and angle-resolved photoluminescence

**Fig. 4:**
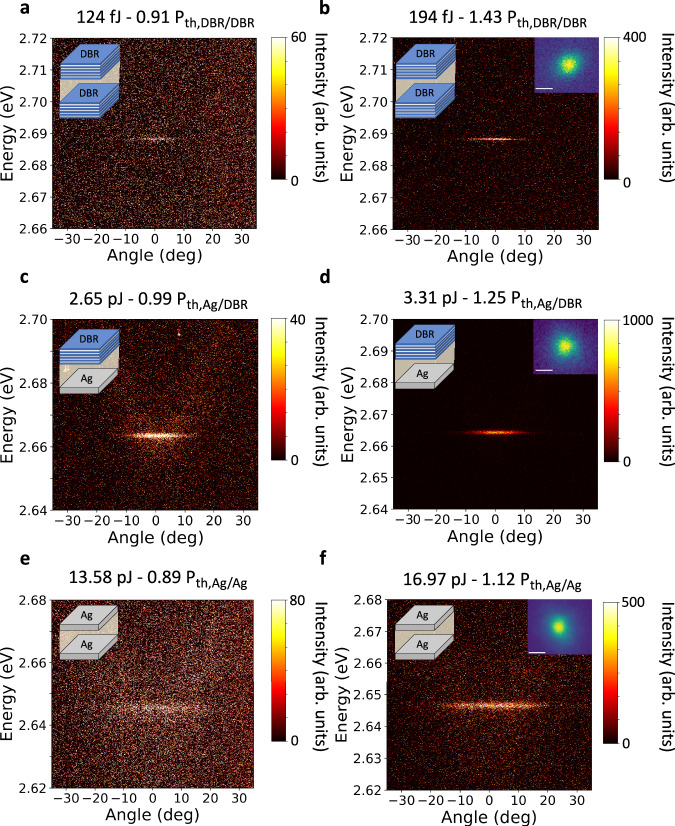
False color maps of angle-resolved emission spectra below and above threshold for focused excitation and different device types. **a**, **b** DBR/DBR microcavity. **c**, **d** Ag/DBR microcavity. **e**, **f** Ag/Ag microcavity. Excitation just below (**a**, **c**, **e**) and above (**b**, **d**, **f**) the lasing threshold. The insets in (**b**, **d**, **f**) show the corresponding real space emission; scale bars, 2.5 μm. The excitation polarization was vertical (${\phi }_{{{{\rm{pump}}}}}=0^\circ$). An intensity color scale is given on the right-hand side of each panel.

Next, we show how focusing the excitation beam to match the size and location of local emission spots in the Schlieren cavity enables spectral and spatial selection of localized polariton lasing modes (*d*_pump_ ~ 3 μm). For this, the angular distribution of the emission from the DBR/DBR cavity was measured via Fourier plane imaging while pumping at ${\phi }_{{{{\rm{pump}}}}}=0^\circ$. When exciting below the polariton lasing threshold, the emission was weak and its angular dispersion was flat (Fig. 4a), indicating strong confinement of polaritons and resulting mode discretization^32–34^. Above threshold, however, the emission increased nonlinearly, reduced in linewidth to below the resolution of our optical setup (300 μeV), and blue-shifted (Fig. 4b). Above threshold, the emission also shows spatial coherence as demonstrated by the appearance of interference fringes on a Michelson interferometer in retro-reflector configuration (Supplementary Fig. 8). These features are hallmarks of the onset of polariton lasing^8,9,35^.

Fig. 4c shows the angle-resolved emission of a hybrid Ag/DBR cavity at an excitation energy just below the threshold between the linear and super-linear regimes; both a very weak parabolic background emission and an intense flat emission at around 2.665 eV are visible. Above threshold, the flat emission overshadows the background (Fig. 4d). The spectra of the purely metallic Ag/Ag cavity are similar to the hybrid Ag/DBR cavity, but the excitation pulse energy required to reach threshold is higher and the emission linewidths are broader due to additional losses induced by the second Ag mirror (Fig. 4e, f). The weak background emission observed for the Ag/DBR and Ag/Ag cavities is associated with non-localized emission emanating from the surroundings of the confined spot; it is more visible for the Ag/DBR and Ag/Ag cavities as the excitation pulse energies used for these are more than one order of magnitude higher than for the DBR/DBR cavity. For all three cavity types, no emission was observed when the excitation polarization was set to ${\phi }_{{{{\rm{pump}}}}}=90^\circ$ while keeping the same excitation spot on a 0°-oriented domain; this illustrates how the strong anisotropy of the system enables polarization selection.

### Hallmarks of polariton lasing

**Fig. 5:**
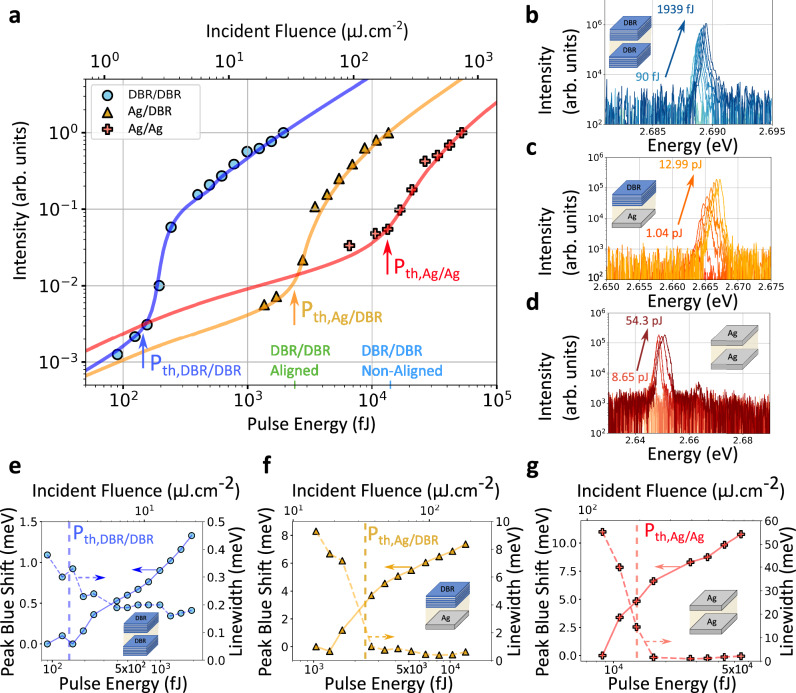
Analysis of nonlinear intensity increase, blueshift, and linewidth reduction of the emission for the different cavity types. **a** Integrated emission intensity versus incident excitation pulse energy and incident fluence for the DBR/DBR (blue symbols), Ag/DBR (orange symbols), and Ag/Ag (red symbols) cavities. Arrows indicate the corresponding polariton lasing thresholds (P_th_) as determined by fitting the measured emission intensity to a kinetic model. The short vertical lines on the x-axis indicate the polariton lasing threshold pulse energies for an aligned DBR/DBR (green) and an isotropic DBR/DBR (blue) cavity without Schlieren texture (values taken from in ref. ^19^). Emission spectra collected over an angular range $\theta \in [-2^\circ ;2^\circ ]$ for increasing excitation pulse energy, transitioning from spontaneous LP emission to polariton lasing, for the DBR/DBR (**b**), the Ag/DBR (**c**), and the Ag/Ag (**d**) cavity. LP linewidth (dashed line) and blue-shift of LP peak (solid line) for the DBR/DBR (**e**), the Ag/DBR (**f**), and the Ag/Ag (**g**) microcavity. The dashed vertical lines indicate the polariton lasing thresholds for each cavity as determined in (**a**). In all cases, the excitation polarization was set to ${\phi }_{{{{\rm{pump}}}}}=0^\circ$.

Fig. 5a shows the emission intensity versus the incident excitation pulse energy for all samples, in each case integrated over ${{{\rm{\theta }}}}\in [-2^\circ ;2^\circ ]$. The PL spectra used for the integration are shown in Fig. 5b–d. For all samples, a clear, super-linear increase in intensity is observed above a specific threshold excitation pulse energy *P*_th_. The observation of organic polariton lasing from vertical cavities with purely metallic mirrors is particularly noteworthy; due to plasmonic losses, polariton lasers have so far generally relied on DBR mirrors or hybrid metal cavities partially supported by DBRs^36–39^.

All three cavity types show a reduction in linewidth and a blueshift of the emission peak, both hallmarks of polariton lasing (Fig. 5e–g). The ~40 meV reduction in linewidth seen for the Ag/Ag cavity is remarkable; reductions reported in other studies are on the order of a few meV^14,15,32^. In addition, the blueshift of the emission peak increases from ~2 meV for the DBR/DBR cavity to ~12 meV for the Ag/Ag cavity, which again is more than twice as large as the values reported for other DBR/DBR cavities^14,15,32^. The characteristic blueshifts in organic polariton condensates were recently shown^35^ to originate from quenching of the Rabi-splitting

1 $${{\hslash }}{\Omega }_{R}={{\hslash }}{\Omega }_{0}\sqrt{1-\frac{2({n}_{x}+{n}_{p})}{{n}_{0}}}$$

where $\hslash {\Omega }_{0}$ is the vacuum Rabi-splitting, ${n}_{{{{\rm{x}}}}}$ the number of excitons, ${n}_{{{{\rm{p}}}}}$ the number of polaritons, and ${{{n}}}_{0}$ the total number of molecules participating in strong coupling, as the Pauli-blocking principle prevents occupied states from being filled twice (a counter-intuitive observation that stems from the non-bosonic nature of excitons at low excitation densities). The increase in the peak blueshift observed from DBR/DBR to Ag/DBR to Ag/Ag is thus a result of both the increase in ${n}_{{{{\rm{x}}}}}+{n}_{{{{\rm{p}}}}}$ due to the increase in polariton lasing threshold and the increase in $\hslash {\Omega }_{0}$ due to the use of metallic mirrors^6^. Containing the electric field more fully inside the active layer with such mirrors is also a desirable lasing feature that has only been observed for a bound state in the continuum (BIC) so far^40^.

### Lasing performance and coherence

The dependence of integrated emission on excitation energy was analyzed further by fitting a kinetic model for the time evolution of the populations of reservoir excitons, *n*_R_, and LP, *n*_LP_, to our data. The master equations for our system are

2 $$\frac{{{{\rm{d}}}}{n}_{{{{\rm{R}}}}}}{{{{\rm{dt}}}}}=\left(1-\frac{{n}_{{{{\rm{R}}}}}}{{{{N}}}_{0}}\right){{P}}\left({{{\rm{t}}}}\right)-\frac{{n}_{{{{\rm{R}}}}}}{{\tau }_{{{{\rm{R}}}}}}-{k}_{{{{\rm{B}}}}}{n}_{{{{\rm{R}}}}}^{2}-\frac{{W}_{{{{\rm{ep}}}}}}{d}{n}_{{{{\rm{R}}}}}{n}_{{{{\rm{LP}}}}}$$

3 $$\frac{{{{\rm{d}}}}{n}_{{{{\rm{LP}}}}}}{{{{\rm{dt}}}}}={W}_{{\!{{\rm{ep}}}}}{n}_{{{{\rm{R}}}}}{n}_{{{{\rm{LP}}}}}-\frac{{n}_{{{{\rm{LP}}}}}}{{\tau }_{{{{\rm{LP}}}}}}+d{{\cdot }}f\frac{{n}_{{{{\rm{R}}}}}}{{\tau }_{{{{\rm{R}}}}}}$$

where ${{{N}}}_{0}$ is the excitation density in the PFO film^41^ (~7 × 10^20^ cm^−3^), ${\tau }_{{{{\rm{R}}}}}$ is the reservoir exciton lifetime (measured at 450 ps, in line with previous reports^42^), *d* is the active layer thickness, and ${\tau }_{{{{\rm{LP}}}}}$ is the LP lifetime. *P*(t) is the temporal profile of the pump pulse, approximated as $P\left({{t}}\right)=2{P}_{{{{\rm{eff}}}}}\exp (-{{{t}}}^{2}{\left(2{\sigma }^{2}\right)}^{-1})$ with a ${\rm{FWHM}}=2\sqrt{2{{\mathrm{ln}}}2}\sigma=25\,{{{\rm{ps}}}}$ and ${P}_{{{{\rm{eff}}}}}={P}_{0}{({dS}{{\hslash }}{\omega }_{{{{\rm{pump}}}}})}^{-1}$ where *S* is the size of the excitation spot, $\hslash {\omega }_{{{{\rm{pump}}}}}=3.49\,{{{\rm{eV}}}}$, ${P}_{0}$ is the pump pulse energy, and the factor 2 in front of ${P}_{{{{\rm{eff}}}}}$ accounts for enhanced absorption due to alignment of the exciton transition dipole moments and pump polarization^18,19^. The free parameters for non-textured cavities, e.g., a macroscopically aligned layer of 15% β*-*phase PFO, are the exciton bimolecular annihilation rate, ${k}_{{{{\rm{B}}}}}$, the exciton reservoir-to-LP resonant scattering rate, ${W}_{{{{\rm{ep}}}}}$, and the fraction of spontaneous scattering from the exciton reservoir to the LP, *f*. The latter can be regarded as the equivalent of the β-factor in conventional photonic lasers, yet its interpretation in lossy systems remains challenging^15^. An estimate for ${\tau }_{{{{\rm{LP}}}}}$ is obtained from the linewidth of sub-threshold LP emission at $\theta=0^\circ$ and is referred to as ${\tau }_{{{{\rm{LP}}}}-\exp }$.

The values of ${k}_{{{{\rm{B}}}}}$ and ${W}_{{{{\rm{ep}}}}}$ fitted for a cavity with a macroscopically aligned layer of 15% β*-*phase PFO (Supplementary Note 5 and Supplementary Fig. 7) are then taken as fixed parameters for the Schlieren cavities in which ${\tau }_{{{{\rm{LP}}}}-{{{\rm{fit}}}}}$ and *f* become the only free parameters in order to relate the reduction in threshold to the narrow emission linewidths measured in the linear regime. Table 1 summarizes the laser performances for the DBR/DBR, Ag/DBR, and Ag/Ag cavities and the corresponding kinetic parameters. The radiative decay times fitted with the kinetic model $({\tau }_{{{{\rm{LP}}}}-{{{\rm{fit}}}}})$ are in close agreement with the decay times extracted from the linewidth of the LP emission below threshold $({\tau }_{{{{\rm{LP}}}}-\exp })$. The spontaneous scattering fraction, *f*, increases with the introduction of the Ag mirrors; *f*_DBR/DBR_ = 0.03, *f*_Ag/DBR_ = 0.17, and *f*_Ag/Ag_ $\approx$ 1.

**Table 1:** Parameters extracted from the kinetic model and polariton lasing performance for the DBR/DBR, the Ag/DBR, and Ag/Ag cavities

Sample	LP radiative decay time from peak width ${\tau }_{{{{\rm{LP}}}}-\exp }$ [fs]	Resonant polariton scattering rate *W*_ep_ [cm^3^ s^−1^]	Spontaneous scattering fraction *f*	Bimolecular annihilation rate ${k}_{{{{\rm{B}}}}}$[cm^3^ s^−1^]	LP radiative decay time from kinetic model ${\tau }_{{{{\rm{LP}}}}-{{{\rm{fit}}}}}$ [fs]	Incident threshold pulse energy *P*_th_
DBR/DBR	1320	2.6 × 10^−6^	0.03	7.5 × 10^−9^	660	136 fJ
Ag/DBR	83	2.6 × 10^−6^	0.17	7.5 × 10^−9^	55	2.67 pJ
Ag/Ag	16.7	2.6 × 10^−6^	$\approx$1	7.5 × 10^−9^	26	15.2 pJ

By fitting the experimental data to the kinetic model, a polariton lasing threshold of ${P}_{{{{\rm{th}}}},{{{\rm{DBR}}}}/{{{\rm{DBR}}}}}=136\,{{{\rm{fJ}}}}$ is obtained for the DBR/DBR cavity. This is the lowest threshold pulse energy reported for any organic polariton or vertical cavity surface emitting photon laser to date. It is more than 16 times lower than the previous record^19^, achieved for a DBR/DBR cavity with an active layer of macroscopically aligned 15% β-phase PFO (${P}_{{{{\rm{th}}}},{{{\rm{Aligned}}}}}=2.23\,{{{\rm{pJ}}}}$).

Relative to a DBR/DBR cavity with an active layer of macroscopically aligned 15% β-phase PFO, the *Q*-factor of the cavity with the Schlieren textured active layer increased approximately 10-fold (*Q*_Aligned-exp_ ~ 530 vs *Q*_DBR/DBR-exp_ ~ 5300; values estimated from the width of the subthreshold LP spectrum of the respective cavities). We attribute this to a combination of two effects: Firstly, in-plane confinement leads to a direct increase in *Q*-factor (~2-fold increase) and secondly, the reduction in mode volume confines the mode to a cavity region where the height is constant (leading to a ~4–5-fold increase in *Q*-factor, see Supplementary Note 7). By contrast, the Ag/DBR cavity only shows the direct ~2-fold enhancement in Q-factor associated with in-plane confinement. This is consistent with our TMCs, which show that upon introduction of the bottom Ag mirror, metallic losses become dominant over losses induced by the sample roughness (Supplementary Note 7). For the Ag/Ag cavity, losses increase further when the top Ag mirror is evaporated directly on the rough polymer layer as the metal film formation is prone to nanocluster formation.

## Discussion

We demonstrated how the introduction of local dipole orientation and a rough topography in a Schlieren textured LCCP cavity via a simple heating and quenching step can drastically enhance polariton lasing performance. The alignment of the sub-domains in the Schlieren cavity enables the formation of anisotropic polaritons which are then strongly confined by both the boundaries with other domains and the intra-cavity roughness. The resulting emission patterns are unique thanks to the intrinsic randomness of the in-plane confinement. The confinement effect allowed polariton lasing with a threshold down to ${P}_{{{{\rm{th}}}},{{{\rm{DBR}}}}/{{{\rm{DBR}}}}}=136\,{{{\rm{fJ}}}}$ per pulse for our DBR/DBR cavity, which represents an impressively low threshold for this kind of system; see in ref. ^19^ for a review of recently reported records in polariton lasing threshold. Furthermore, the experiments on the cavity containing both a Schlieren textured region and a region with uniformly aligned macro-domains revealed that lasing occurs from spatially localized traps in both cases (Supplementary Fig. 6). However, in the Schlieren textured cavities, the density of these lasing spots appears to be lower, and the spots are more well-defined, which may be useful for future devices, especially for electrical injection pumped polariton lasing.

Our findings thus demonstrate that contrary to what one might expect, the presence of roughness can greatly enhance the performance of polariton lasers. The threshold pulse energy in our system corresponds to an incident pulse fluence of ${F}_{{{{\rm{inc}}}}}$= 1.94 μJ cm^−2^. (We have refrained from calculating the absorbed pump fluence because the inhomogeneous nature of the Schlieren textured films prevents an accurate estimate of absorption via TMC.) A direct comparison of this value to the threshold fluence of polariton lasers with homogeneous active layers is however not particularly meaningful due to the strongly reduced mode volume in our Schlieren textured device. In general, whether the pulse fluence or the absolute pulse energy at threshold is the more important figure of merit, depends on the intended application of the laser. The narrow LP linewidth of ~500 μeV in the DBR/DBR cavity suggests a LP lifetime of >1 ps, which could be used to coherently address polaritons before their decay through the DBR mirrors. From a broader viewpoint, the Schlieren cavities and in general cavities with a tunable roughness also represent an opportunity to bridge the field of random and polariton lasers as the high degree of disorder inside the Schlieren texture could lead to Anderson localization of polaritons^43–47^, i.e., a situation where a high number of scattering events (i.e., a short optical free path) compared to the effective wavevector would halt the propagation of polaritons. For instance, tuning of the cavity topography might allow to observe a transition between a propagating and halted flow of polaritons.

The dramatic improvement in lasing performance enabled by the Schlieren texture further permitted the fabrication of polariton lasers with conductive metallic mirrors. Use of metallic mirrors is often considered a prerequisite for efficient current injection in future electrically driven organic polariton lasers. However, despite the benefits offered by the reduced penetration depth of metallic mirrors and the resulting smaller mode volume, achieving polariton lasing in a metal-metal cavity has long been considered impossible due to the high optical losses associated with metallic mirrors. Recently, some notable progress has been made on metal cavity polariton lasers, with demonstrations of thermalization of the LP in a hybrid DBR-protein-metal cavity^36^ and lasing in a metal-metal cavity containing thick single-crystal layers (thickness > 2 μm)^48^. However, these structures exhibited either a high threshold (8 nJ per pulse^36^) or involved challenging active layer fabrication^48^.

Future studies on fundamental aspects of polariton lasing as well as the development of new applications based on these phenomena will benefit from our novel bottom-up device platform in which changes in photonic environment allow to tune blueshift over one order of magnitude (from 1 to 10 meV) and giant reductions in linewidth are accessible (up to 40 meV). We anticipate that the wide breadth of LCCPs^49^ and recent advances offered by patternable alignment layers (using either photomasks^50^ or direct two-photon laser writing^27^) will allow to reproduce and optimize the features of the Schlieren texture, e.g., to explore topological phenomena^51^, further optimize performance, and add new functionalities such as polarization sensitivity to polariton based single photon detectors^13^.

## Methods

### Materials

PFO was supplied by the Sumitomo Chemical Company, Japan, and used as received. The peak molecular weight was ${M}_{{{{\rm{pPFO}}}}}=50\times {10}^{3}$ g mol^−1^. For the dielectric mirrors, Ta_2_O_5_ and SiO_2_ were sputtered from >99.99% oxide targets (Angstrom Engineering). For the metal mirrors, silver and aluminum pellets (99.99%, Kurt J. Lesker company) were thermally evaporated. The substrates used were display-grade glass (Eagle XG, Howard Glass), sized 24 x 24 mm^2^.

### Microcavity fabrication

The DBR/DBR cavity contains a 132 nm-thick PFO film with 10.5 Ta_2_O_5_/SiO_2_ (75 nm/51.20 nm) pairs on both sides. From the measurements in ref. ^19^, which use a similar but unpatterned cavity, we estimate that for this configuration we get ${{\hslash }}{\Omega }_{1-{{{\rm{DBR}}}}/{{{\rm{DBR}}}}}\approx 0.5\,{{{\rm{eV}}}}$ and ${{\hslash }}{\Omega }_{2-{{{\rm{DBR}}}}/{{{\rm{DBR}}}}}\approx 0.05\,{{{\rm{eV}}}}$. The Ag/DBR cavity contains a 95 nm-thick PFO film with 100 nm Ag on one side and 10.5 Ta_2_O_5_/SiO_2_ pairs on the other. The Ag/Ag cavity contains a 170 nm-thick PFO film with 100 nm Ag on one side and 60 nm Ag on the other; this second-order cavity design for the Ag/Ag cavity was found to increase the *Q*-factor in these cavities (see Supplementary Fig. 9). The corresponding Rabi-splittings were calculated using a similar enhancement factor as the one observed between DBR/DBR (ref. ^19^) and Al/Al (ref. ^50^) cavities yielding ${{\hslash }}{\Omega }_{1-{{{\rm{Ag}}}}/{{{\rm{Ag}}}}}\approx$ 1.4 eV and ${{\hslash }}{\Omega }_{2-{{{\rm{Ag}}}}/{{{\rm{Ag}}}}}\approx 0.14\,{{{\rm{eV}}}}$. The Rabi-splittings for the Ag/DBR cavity were then approximated using an evenly weighted sum of the DBR/DBR and the Ag/Ag values to give ${{\hslash }}{\Omega }_{1-{{{\rm{Ag}}}}/{{{\rm{DBR}}}}}\approx$ 0.95 eV and ${{\hslash }}{\Omega }_{2-{{{\rm{Ag}}}}/{{{\rm{DBR}}}}}\approx$ 0.1 eV. For metal mirrors, 1 nm Al (as seed layer) was deposited by electron beam physical vapor deposition and Ag was deposited by thermal evaporation in a vacuum chamber (Angstrom EvoVac) at a base pressure of 1 × 10 − 7 mbar. Al was used as a wetting layer to improve percolation and optical quality of the thin Ag films. SiO_2_ and Ta_2_O_5_ were deposited by radiofrequency magnetron sputtering at a base pressure of 10^−7 ^Torr, using 18 standard cubic centimeters per minute (sccm) Argon flow at 2 mTorr process pressure and 18 sccm Argon together with 4 sccm Oxygen flow at 4 mTorr process pressure for SiO_2_ and Ta_2_O_5_, respectively. The additional oxygen flow during Ta_2_O_5_ deposition prevents the formation of unwanted sub-oxides. A layer of PFO was spin-coated using 24 mg mL^−1^ PFO in toluene solution for the DBR/DBR cavity, 16 mg mL^−1^ for the Ag/DBR cavity, 28 mg mL^−1^ for the Ag/Ag cavity, for 1 min at a speed of 2000 rpm, with an initial acceleration of 1000 rpm s^−1^. Next, the sample was placed on a precision hotplate (Präzitherm, Gestigkeit GmbH) in an inert environment and the temperature was raised from 25 °C to 160 °C at a rate of approximately 30 °C min^−1^. The upper temperature was then held for 10 min, followed by rapid quenching to room temperature by placing the sample on a metallic surface to induce the nematic phase Schlieren texture in the PFO film. Subsequently, approximately 15% β*-*phase fraction was induced in the by exposing the films to a saturated toluene vapor environment for 24 h. The thicknesses of the films and thus of the active layers in the final cavity were controlled using a profilometer (Dektak, Bruker) on simultaneously prepared reference samples. Finally, the top DBR or Ag mirror was deposited following the same process described above. AFM measurements were performed using a JPK Bruker NanoWizard 4 mounted on a Nikon Eclipse Ti2 inverted microscope.

### Angle-resolved PL measurements

PL spectra were measured using Fourier imaging spectroscopy by imaging the back focal plane of a 40× objective (numerical aperture 0.75, Nikon Plan Fluor), set up on a commercial inverted microscope stand in reflection configuration (Nikon Eclipse Ti2-E). The third harmonic generation (THG) output of a diode-pumped Nd:YAG laser system (PL2210A, Ekspla), with wavelength 355 nm, repetition rate 250 Hz, and pulse duration 25 ps was used for excitation. Linear polarization of the pump was set by placing a Glan-Taylor polarizer (GT10-A, Thorlabs) before the objective. This polarization was rotated using a zero-order half-wave plate (46–549, Edmund Optics). For the wide spot excitation, the diameter of the Gaussian beam at the sample plane was measured to be d_pump_ ~ 150 μm, while for the small spot excitation, the diameter was reduced down to d_pump_ ~ 3 μm to match the size of the localized emission centres in the Schlieren texture. The emitted light was directed towards the entrance of a spectrograph (Shamrock SR-500i-D2-SiL, Andor) equipped with an 1800 lines mm^−1^ grating blazed at 500 nm, and the PL spectra were imaged on an EM CCD camera (Newton 971, Andor) providing a spectral resolution of 40 pm (~300 μeV at 2.67 eV). The spatial coherence measurements presented in the Supplementary Information were performed using a Michelson interferometer in the retro-reflector configuration and the resulting interferograms were imaged on an sCMOS camera (ORCA-Flash 4.0, Hamamatsu).

### Finite-difference time-domain (FDTD) simulations

FDTD simulations of the in-plane component of ${\left|{{{\boldsymbol{E}}}}\right|}^{2}$ were performed to reproduce the localization of exciton-polaritons inside the active layer. The simulations were performed using the FDTD 3D Electromagnetic Simulator from Lumerical-Ansys.

## Supplementary information

Supplementary Information

Transparent Peer Review file

## Data Availability

The data generated in this study are openly available via the St Andrews Research Portal at 10.17630/7382ac95-03eb-4456-ad70-2f3fd1aeae88.
