# Supplementary material for: Schlieren texture and topography induced confinement in an organic exciton-polariton laser
Source: Nat Commun. 2025 Jan 18;16:811. doi: 10.1038/s41467-025-55875-1 (PMC11743153; doi:10.1038/s41467-025-55875-1)
Supplement: Supplementary file 1 — Supplementary Information [file 41467_2025_55875_MOESM1_ESM.pdf]

Supplementary Information

Schlieren texture and topography induced confinement in an organic exciton-polariton laser

Florian Le Roux[1*], *Andreas Mischok*[1], *Francisco Tenopala-Carmona*[1], *Malte C. Gather*[1,2*]

[1] Humboldt Centre for Nano- and Biophotonics, Department of Chemistry, University of Cologne, Greinstr. 4-6, 50939 Köln, Germany

[2] Organic Semiconductor Centre, SUPA School of Physics and Astronomy, University of St Andrews, St Andrews, KY16 9SS, UK

Supplementary Note 1 - Optical constants for aligned PFO β-phase

The optical constants and PL spectrum of macroscopically aligned 15% β-phase PFO depicted in **Supplementary Figure 1**a, b show a strong preferential alignment of the polymer along the y-direction and a well-resolved vibronic progression of the emission from β-phase PFO, respectively emission.

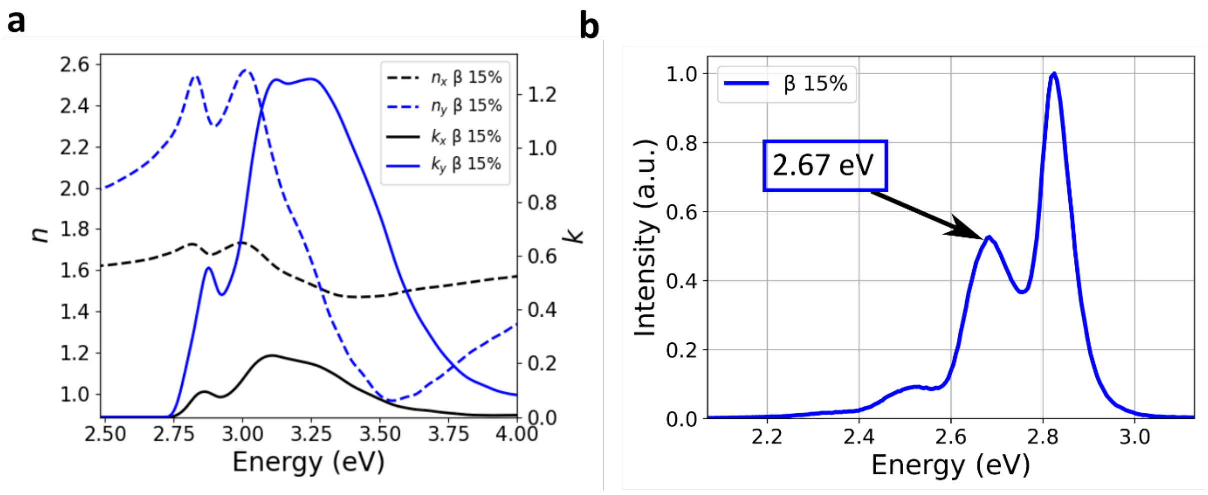

Supplementary Figure 1. Photophysical properties of aligned PFO β-phase. a, In-plane optical constants of macroscopically aligned 15% β-phase PFO along the direction of alignment (y, blue lines) and perpendicular to the alignment (x, black lines), showing the extinction coefficient, $\boldsymbol{k_x}$ and $\boldsymbol{k_y}$ (solid lines), and refractive index, $\boldsymbol{n_x}$ and $\boldsymbol{n_y}$ (dashed lines). **b**, PL spectrum for a spin-coated and aligned PFO thin film containing 15% β-phase. (Reproduced with permission from Ref. 3.)

Supplementary Note 2 - Emission from Schlieren textured cavity under polarized excitation

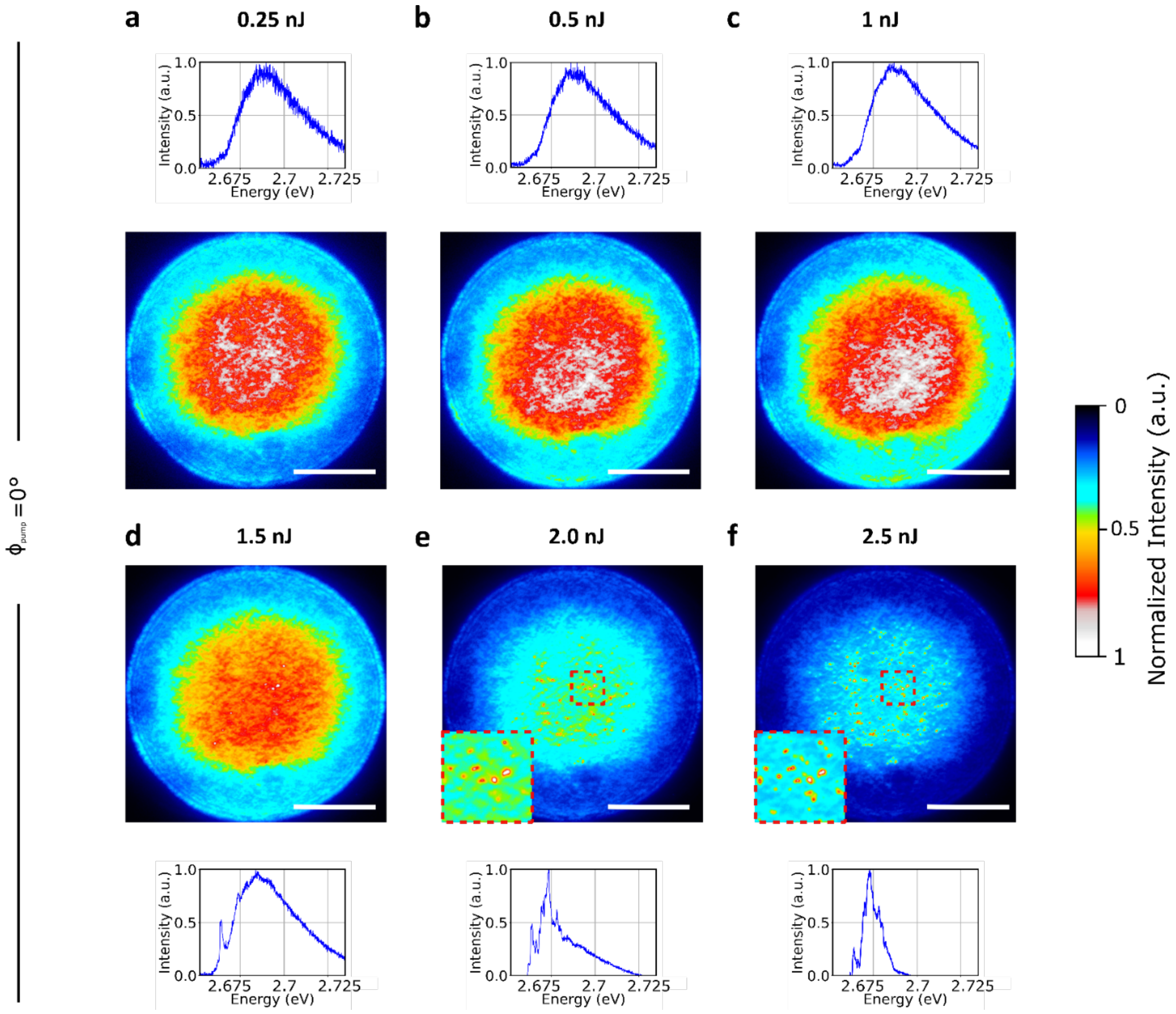

Supplementary Figure 2. Spatially and spectrally resolved emission from the Schlieren textured DBR/DBR cavity under non-resonant optical pumping with ϕ_{pump} = 0°. Spatially and spectrally resolved emission from the DBR/DBR cavity for increasing excitation pulse energies; a) 250 pJ, b) 500 pJ, c) 1 nJ, d) 1.5 nJ, e) 2 nJ, f) 2.5 nJ per pulse. The insets in e and f show magnifications of the red dashed rectangles in the corresponding main panels. Scale bars: 50 µm.

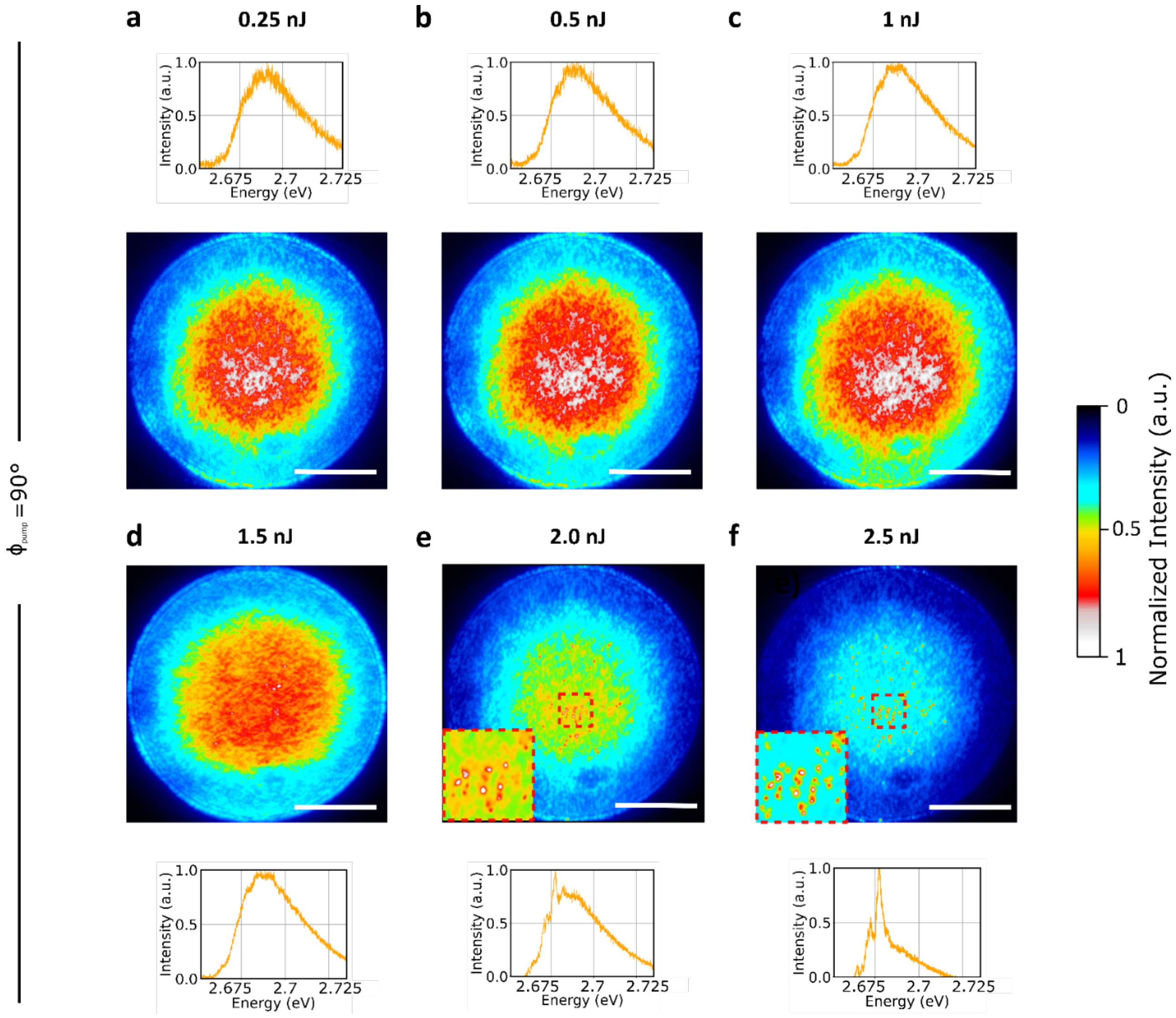

Supplementary Figure 3. Spatially and spectrally resolved emission from the Schlieren textured DBR/DBR cavity under non-resonant optical pumping with $\phi_{\text{pump}} = 90°$. Spatially and spectrally resolved emission from the DBR/DBR cavity for increasing excitation pulse energies; a) 250 pJ, b) 500 pJ, c) 1 nJ, d) 1.5 nJ, e) 2 nJ, f) 2.5 nJ per pulse. The insets in e and f show magnifications of the red dashed rectangles in the corresponding main panels. Scale bars: 50 µm.

Supplementary Note 3 - In-plane confinement of polaritons in the Schlieren textured cavity

FDTD simulations of the in-plane component of $|\mathbf{E}|^2$ were performed to reproduce the localization of exciton-polaritons inside the active layer. The simulations were performed using the FDTD 3D Electromagnetic Simulator from Lumerical-Ansys[1].

Supplementary Figure 4a shows the vertical structure of the DBR/DBR cavity used for the simulation. The in-plane simulated surface was 13.97 μm wide by 13.97 μm long, in correspondence with the optical polarized micrograph shown in Supplementary Figure 3b. The spatially-resolved PL obtained using a 150 μm wide polarized excitation spot with a pulse energy of 1.5 nJ and ϕ_{pump}= 0° is also shown in Supplementary Figure 4c with the high intensity emission centre clearly visible.

Local Transition Dipole Moment Orientation

It has been shown that the combination of cross-polarized microscopy and polarized PL is an efficient method to lift the inherent degeneracy that exist between orthogonal directions in cross-polarized microscopy[2]. PL measurements represented in Supplementary Figure 4d for ϕ_{pump}= 0° (left, red) and ϕ_{pump}= 90° (right, blue) at an excitation energy of 500 pJ were therefore recorded in order to delineate the orientation of the local transition dipole moments.

Supplementary Figure 4e shows a comparison between the emission intensities obtained for ϕ_{pump} = 0° (red) and ϕ_{pump} = 90° (blue). Since both images are 8-bit grayscale, the emission intensity of each pixel $I_{x,y}$ is an integer between 0 and 255. The pixel color $C_{x,y}$ in Figure S3e is obtained as follows: If $I_{x,y\text{-}0°} > 130$ and $I_{x,y\text{-}90°} < 130$, $C_{\text{x,y}}$ is set to red, which means that this location (x,y) is dominated by emission from transition dipole moments closer to 0 than 90° (we call this red domain the 0° domain in the following). Conversely, if $I_{x,y\text{-}0°} < 130$ and $I_{x,y\text{-}90°} > 130$, $C_{x,y}$ is set to blue, which means that this location (x,y) is dominated by emission from transition dipole moments closer to 90° than 0°(we call this blue domain the 90° domain in the following). Finally if both $I_{x,y\text{-}0°} > 130$ and $I_{x,y\text{-}90°} > 130$, $C_{x,y}$ is green and the pixel is not clearly dominated by either orientation.

Domains resulting from this procedure are mostly complimentary with large regions dominated by either the 0° or 90° orientation. The final delimitation between 0° or 90° domains is obtained

by comparing the domains in Supplementary Figure 3e to the polarized micrograph in Supplementary Figure 3b which enables the attribution of the green domains to either the 0° or 90° domains. The left-most panel in Supplementary Figure 4f shows the final 0° and 90° domains.

The local transition dipole moment orientation $T_{x,y}$ is then calculated using the intensity of the corresponding pixel $M_{x,y}$ in the polarized micrograph: a dark state with 0 intensity is observed for orientation of the local transition dipole moment parallel to either the polarizer or analyzer, a bright state with 255 intensity is observed when the transition dipole moment is at 45° relative to either the polarizer or the analyzer. A simplified linear procedure for calculating $T_{x,y}$ is then as follows: if (x,y) belongs to the 0° domain, then $T_{x,y}$ belongs to [0°,45°] and $T_{x,y} = \frac{45 \times \mathrm{M_{x,y}}}{255}$; conversely, if (x,y) belongs to the 90° domain, then $T_{x,y}$ belongs to [45°,90°] and $T_{x,y} = 90 - \frac{45 \times \mathrm{M_{x,y}}}{255}$. The final orientations of T are represented by black arrows and shown in the two panels of Supplementary Figure 1g.

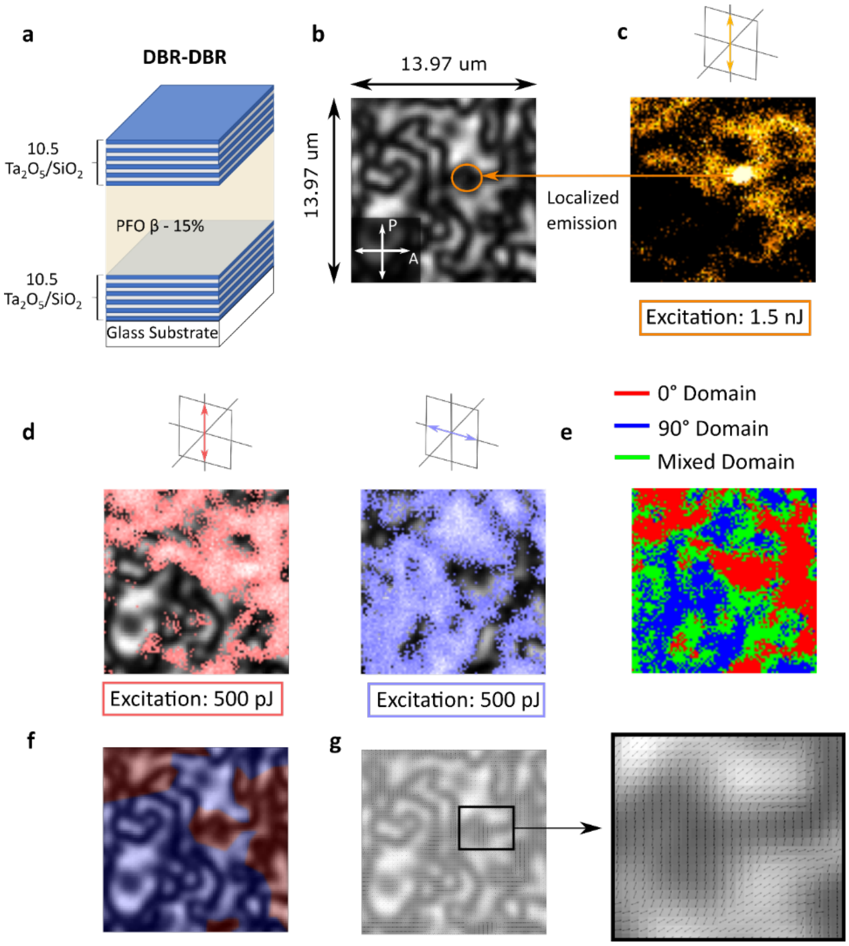

Supplementary Figure 4. Setting up of the FDTD simulation. a, Schematic of the DBR/DBR cavity used as model in the FDTD simulation. **b**, Experimental polarized optical micrograph of the surface of the DBR/DBR cavity recorded with the sample placed between the crossed polarizer (P) and analyzer (A) pair. No light is transmitted when the exciton transition dipole moment lies either parallel to the polarizer or the analyzer, while maximum transmission occurs when the transition dipole moment lies at 45° relative to both the analyzer and polarizer. **c**, Real-space PL image obtained using a 150 µm wide polarized excitation spot with a pulse energy of 1.5 nJ and ϕ_{pump}= 0°. The high intensity spot is clearly visible and its position in b is marked by an orange circle. **d**, Real-space emission images obtained using ϕ_{pump} = 0° (red, left) and ϕ_{pump} = 90° (blue, right) at 500 pJ. The images are overlayed on the polarized optical micrograph from b. **e**, Comparative image obtained following the procedure described in the text showing 0° (red), 90° (blue) or mixed (green) domains. **f**, 0° (red), 90° (blue) domains following comparison of e with the domains of the polarized micrograph in b. **g**, Black arrows representing the local transition dipole moment orientation. The panel on the right shows a zoomed-in version of this image. Both images are overlayed on the polarized optical micrograph from b.

FDTD simulations

The FDTD simulation is passive in the sense that no re-emission is considered once the electric field has been absorbed by one of the elements of the simulation. The optical constants used for the different materials (Ta_2O_5, SiO_2, aligned 15% β-phase PFO) were reproduced from Ref. 3. The active layer was defined as a liquid-crystalline layer in which the local transition dipole moment $T_{x,y}$ can be set by applying the corresponding transformation to the refractive index of the layer.

The simulation monitors the in-plane component of $|\boldsymbol{E}|^2$ inside the active layer for the first 2500 fs. The so-called active region where initial emission is permitted is a 7 μm x 7 μm square in the center of the 13.97 μm x 13.97 μm region of interest to avoid border aberrations that can arise with perfectly matched layers (PMLs) boundary conditions. The emission region is represented by an orange rectangle in Supplementary Figure 1a. Each pixel was given an initial emission power $P_{x,y}(t=0)$ proportional to the square of the dot product between the transition dipole moment orientation $T_{x,y}$ and the excitation polarization (ϕ_{pump} = 0°), simulating initial absorption inside the cavity. Each dipole is given a random phase to prevent interference and broad spectral characteristics resembling a thin-film of PFO in its β-phase, i.e., center energy at 2.67 eV to match the (0-1) vibronic peak of the emission, pulse length of 10 fs and bandwidth of 183 meV.

The resulting $|\boldsymbol{E}|^2$ inside the active layer at multiple time points between 0 fs and 2496 fs is shown in **Supplementary Figure 5**. Confinement of the emission in localized spots is evident in Supplementary Figure 4b and matches the high-intensity PL spot of Supplementary Figure 4c. The combination of cross-polarized microscopy, polarized PL and FDTD simulation forms a precise tool for the characterization of exciton-polaritons localization inside the structure. The simulation also confirms that the mode surface contributing to the mode volume $V_{\text{sim}} = h_{\text{eff}} \times S_{\text{sim}}$, where h_{eff} is an effective height which mainly depends on the penetration depth through the mirrors, is reduced compared with aligned active layers[3]: S_{Aligned} ~20 μm^2 compared with S_{Sim} ~7 μm^2, a nearly three-fold reduction in mode volume between the aligned and textured cavities.

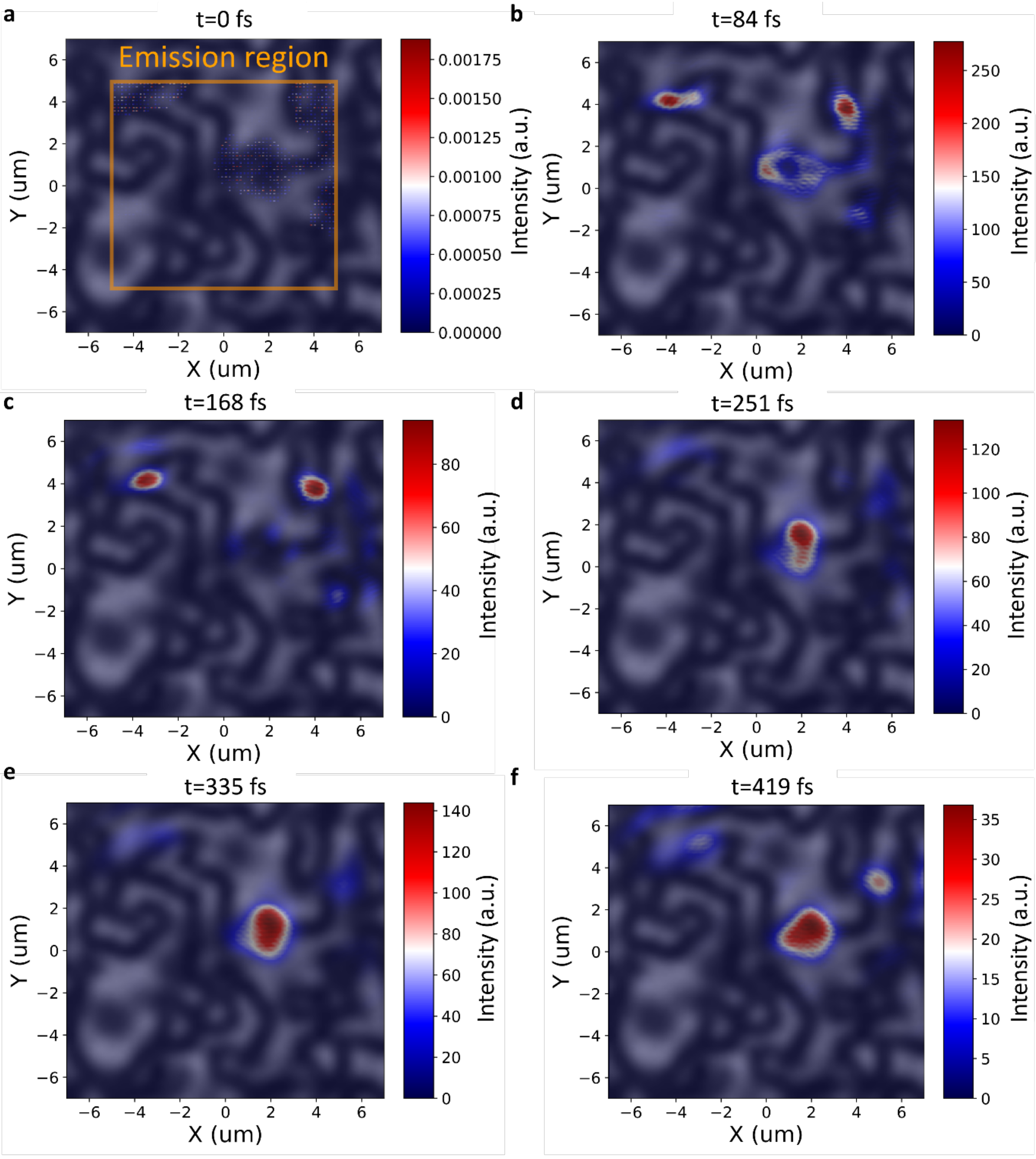

a
t=0 fs
Emission region
Y (um)
X (um)
Intensity (a.u.)
b
t=84 fs
Y (um)
X (um)
Intensity (a.u.)
c
t=168 fs
Y (um)
X (um)
Intensity (a.u.)
d
t=251 fs
Y (um)
X (um)
Intensity (a.u.)
e
t=335 fs
Y (um)
X (um)
Intensity (a.u.)
f
t=419 fs
Y (um)
X (um)
Intensity (a.u.)

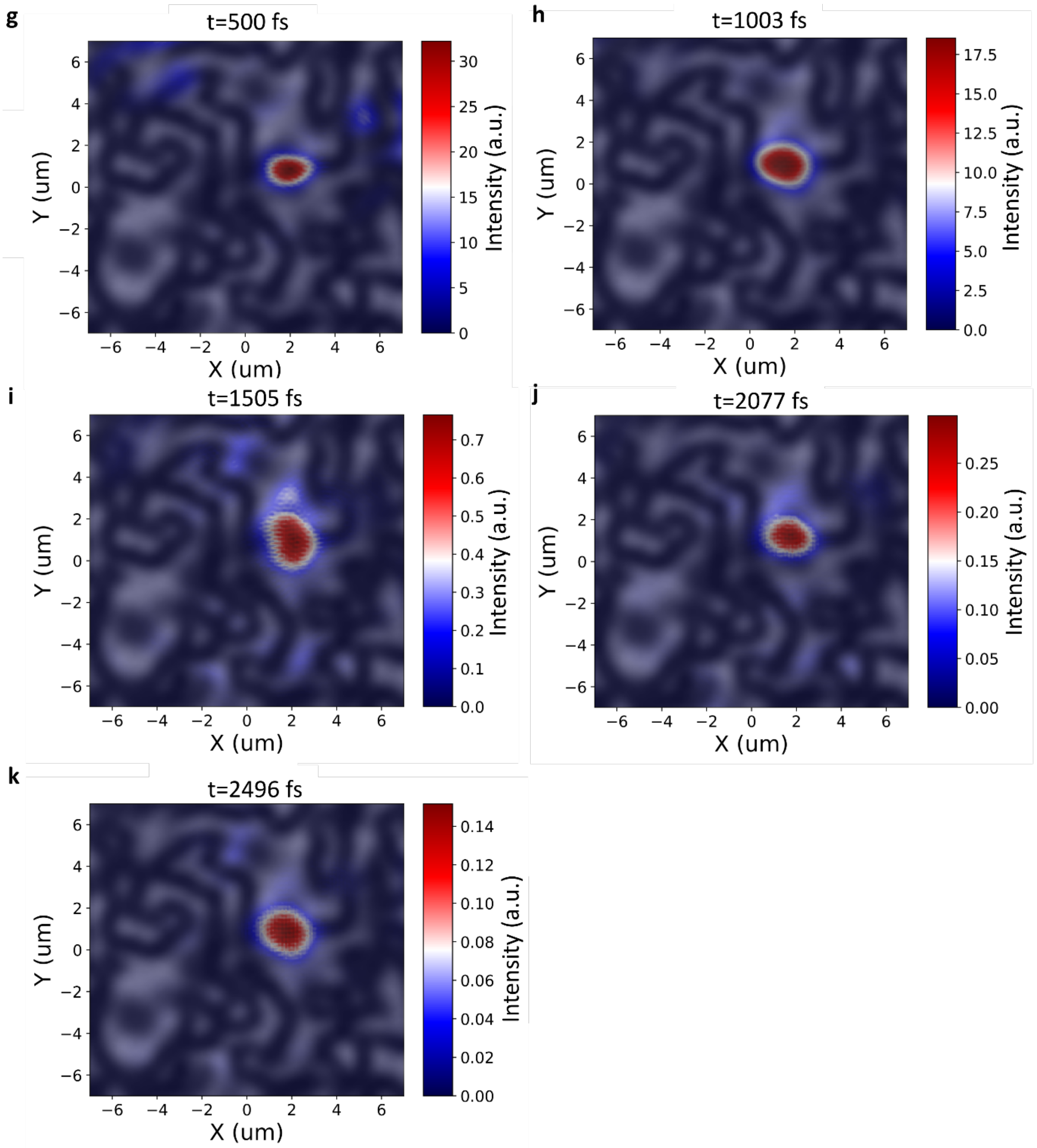

Supplementary Figure 5. In-plane field intensity simulated by FDTD. Two dimensional FDTD calculations of in-plane $|\boldsymbol{E}|^2$ at times a) t = 0 fs, b) t = 84 fs, c) t = 168 fs, d) t = 251 fs, e) t = 335 fs, f) t = 419 fs, g) t = 500 fs, h) t = 1003 fs, i) t = 1505 fs, j) t = 2077 fs and k) t = 2496 fs. The electric field intensity is overlaid on top of the polarized optical micrograph shown in Figure 1a. The simulations were performed following the procedure described in the text. The region inside the orange rectangle corresponds to the initial emission region. Confinement of the electric field intensity is evident and confirms the experimental observation in Figure 1b.

Supplementary Note 4 - The role of intra-cavity topography

As stated in the main text, a 15% β-phase PFO aligned cavity displays a roughness of R_a = 0.88 nm (**Supplementary Figure 6a)**. In Figure 3 of the main text, our FDTD simulations show that a hypothetical cavity with 15% β-phase PFO active layer and an average roughness of R_a = 2.6 nm would support strong in-plane confinement of exciton polaritons. In order to induce this level of roughness experimentally in an otherwise macroscopically aligned cavity, we combined the Schlieren approach from this work with the photoalignment approach from Ref. 3. We first spin-coated a thin layer of the azobenzene molecule SD1 (that also used to induce alignment in Ref. 3) and partially aligned it using a polarized UV light source and a photomask. We then spin-coated the PFO layer on top and aligned it using the same process as for the cavities in the main text. This resulted in a large, aligned domain across the non-masked –and thus UV exposed– part of the sample (showing in black in the polarised optical micrograph, Supplementary Figure 6b), and Schlieren domains for the masked part of the sample where the SD1 was not aligned. The resulting aligned domain now possesses a roughness comparable with the Schlieren texture (R_a = 2.6 nm; AFM measurement, Supplementary Figure 6c). Supplementary Figure 6d and e show the emission observed at the interface between the macroscopically aligned and the Schlieren textured region, using similar excitation conditions as in Figure 2 of the main text. Even at low excitation pulse energies, the emission from the Schlieren region shows a characteristic patterned emission (Supplementary Figure 6d, left), consistent with the observation presented in Figure 2. By contrast, the emission from the aligned region is mostly homogenous at low excitation pulse energies (Supplementary Figure 6d, right). Upon increasing the excitation power, however, confined emission spots appear in both the Schlieren part of the sample and the macroscopically aligned domain (Supplementary Figure 6e). This is in contrast to our earlier observations in Ref. 3 for a macroscopically aligned PFO film with lower roughness and confirms the FDTD simulations presented in Figure 3d of the main text.

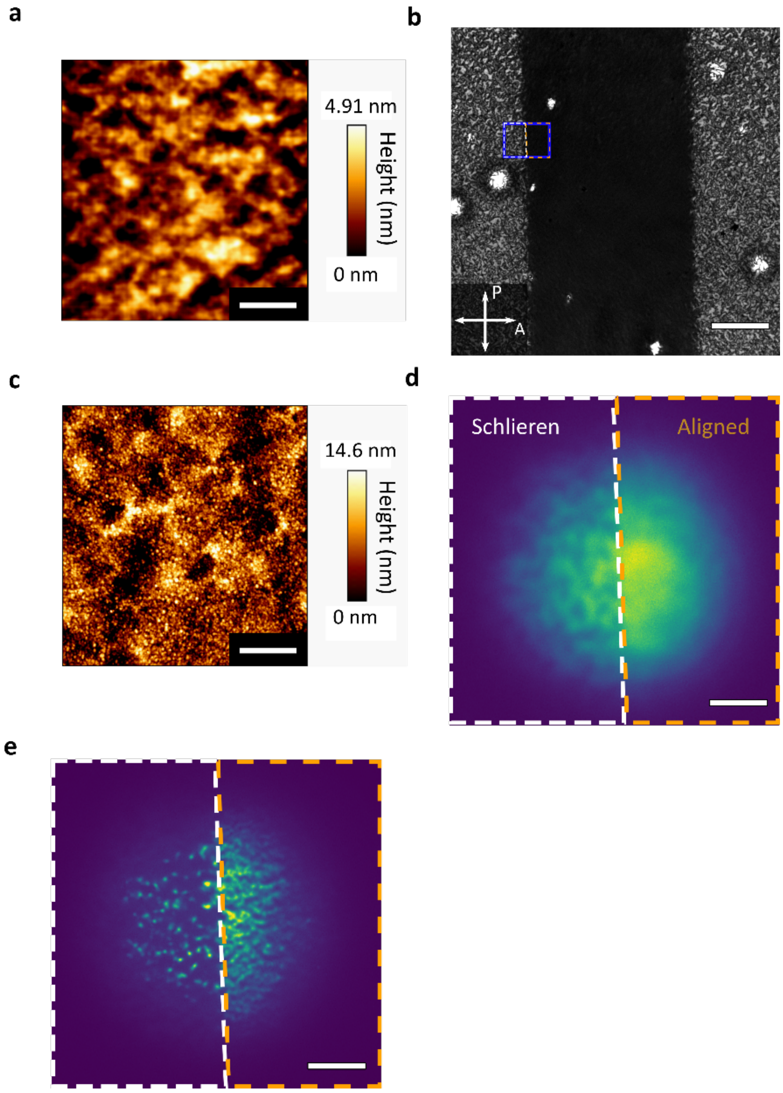

Supplementary Figure 6. The role of intra-cavity topography. a) AFM measurement on a 15% β-phase aligned PFO cavity. The average roughness is R_a = 881 pm. Scale bar, 2μm. b) Polarised optical micrograph of a cavity fabricated according to the description in Section IV using masked alignment of SD1. The blue rectangle marks a border between the Schlieren textured region (left, indicated by white dashes) and the macroscopically aligned domain (right, indicated by orange dashes). c) An AFM measurement performed on the macroscopically aligned domain (region marked in orange in b). The average roughness is R_a = 2.6 nm. d) Emission from Schlieren textured region and the macroscopically aligned domains below threshold (500 pJ). No clear confinement is observed. e) When pumped above threshold (here at 3 nJ), the emission from both regions shows similar confined emission spots, indicative of similar, topography-induced in-plane polariton confinement in each.

Supplementary Note 5 - Kinetic Model for the Aligned and Non-Aligned Cavities

The input-output lasing curves were analysed using a kinetic model derived from the Gross-Pitaevskii equation[4]. The resulting master equation for the polariton population, $n_{\mathrm{LP}}(t)$, and exciton reservoir population, $n_R(t)$, is:

$$\frac{dn_R}{dt} = \left(1 - \frac{n_R}{N_0}\right) P(t) - \frac{n_R}{\tau_R} - k_B n_R^2 - \frac{W_{ep}}{d} n_R n_{LP} \quad (1)$$

$$\frac{dn_{LP}}{dt} = W_{ep} n_R n_{LP} - \frac{n_{LP}}{\tau_{LP}} + df \frac{n_R}{\tau_R} \quad (2)$$

where N_0 is the bare excitation density inside a PFO film (~ 7 × 10^{20} cm^{-3}), τ_{R} the reservoir exciton lifetime (~ 450 ps), d the active layer thickness, τ_{LP} the LP lifetime. The pump term $P(t)$ is a Gaussian pump term, i.e. $P(t) = \boldsymbol{o_a} P_{\mathrm{eff}} \exp(-t^2(2\sigma^2)^{-1})$ with $FWHM = 2\sqrt{2ln2}\sigma = 25$ ps and $P_{\mathrm{eff}} = P_0 \left(dS\hbar\omega_{\mathrm{pump}}\right)^{-1}$ where S is the pumped surface and $\hbar\omega_{pump}$ = 3.49 eV. The free parameters in the model are the exciton bimolecular annihilation rate, k_{B}, the exciton reservoir-to-LP resonant scattering rate, W_{ep}, and the fraction of spontaneous scattering from the exciton reservoir to the LP f. Additionally, the term o_a corresponds to the pumping of the exciton reservoir population according to the dot product between pump polarization and transition dipole moment $\boldsymbol{o_a} = \frac{2\|\boldsymbol{\mu}.\boldsymbol{E}\|^2}{\|\boldsymbol{\mu}\|^2\|\boldsymbol{E}\|^2}$.

The experimental input-output curves for a non-aligned 15% β-phase PFO cavity and an aligned 15 % β-phase PFO cavity and the corresponding fits to the kinetic model are shown in **Supplementary Figure 7** (reproduced with permission from Ref. 3), and results for the aligned cavity are displayed in **Supplementary Table 1**.

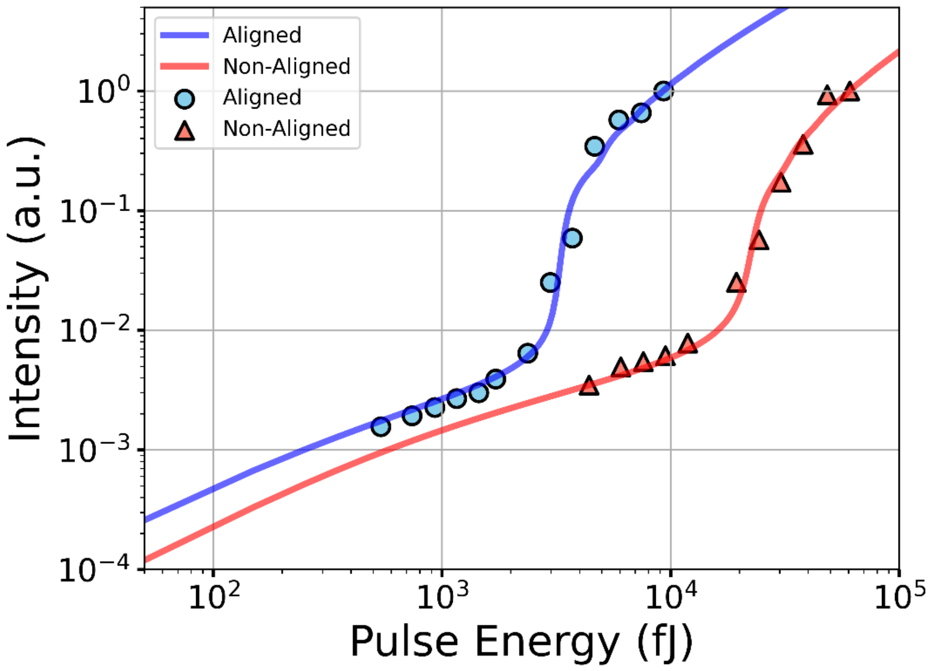

Supplementary Figure 7. Emission intensity for the aligned and non-aligned non-textured cavities. Integrated emission intensity versus incident excitation pulse energy for a DBR/DBR cavity containing a non-aligned (red symbols) and an aligned (blue symbols) layer of 15% β-phase PFO. The data was reproduced with permission from Ref. 3.

Supplementary Table 1. Polariton lasing performance and parameters extracted from the kinetic model for the non-aligned and aligned 15% β-phase PFO cavities (reproduced with permission from Ref. 3).

Sample	Pre-factor o_a	Active layer thickness [nm]	Bimolecular annihilation rate k_B [cm^{3}s^{-1}]	Resonant polariton scattering rate W_{ep} [cm^{3}s^{-1}]	Spontaneous scattering fraction f	LP radiative decay time from peak width τ_{LP-exp} [fs]	Incident threshold pulse energy P_{th}
Non-Aligned (DBR/DBR)	1	155	7.5 x 10^{-9}	1.2 x 10^{-6}	0.080	132	14.50 pJ
Aligned (DBR/DBR)	2	130	7.5 x 10^{-9}	2.6 x 10^{-6}	0.031	132	2.23 pJ

Supplementary Note 6 - Spatial Coherence of the Emission

Spatial coherence of the emission above threshold is a signature of polariton lasing. **Supplementary Figure 8** shows interferometry measurements performed on the different cavities using a Michelson interferometer in the retroreflector configuration[6,7]. The images to the left of the "+" sign show an example of the recorded image obtained by blocking one of the two arms of the interferometer, the images to the right of the "+" sign and thus to the left of the "=" sign show the mirror image obtained by freeing the blocked arm and blocking the other arm of the interferometer. Finally, the images to the right of the "=" sign displays clear fringes obtained through interference of the two paths by having both arms freed, showing clear spatial coherence above threshold.

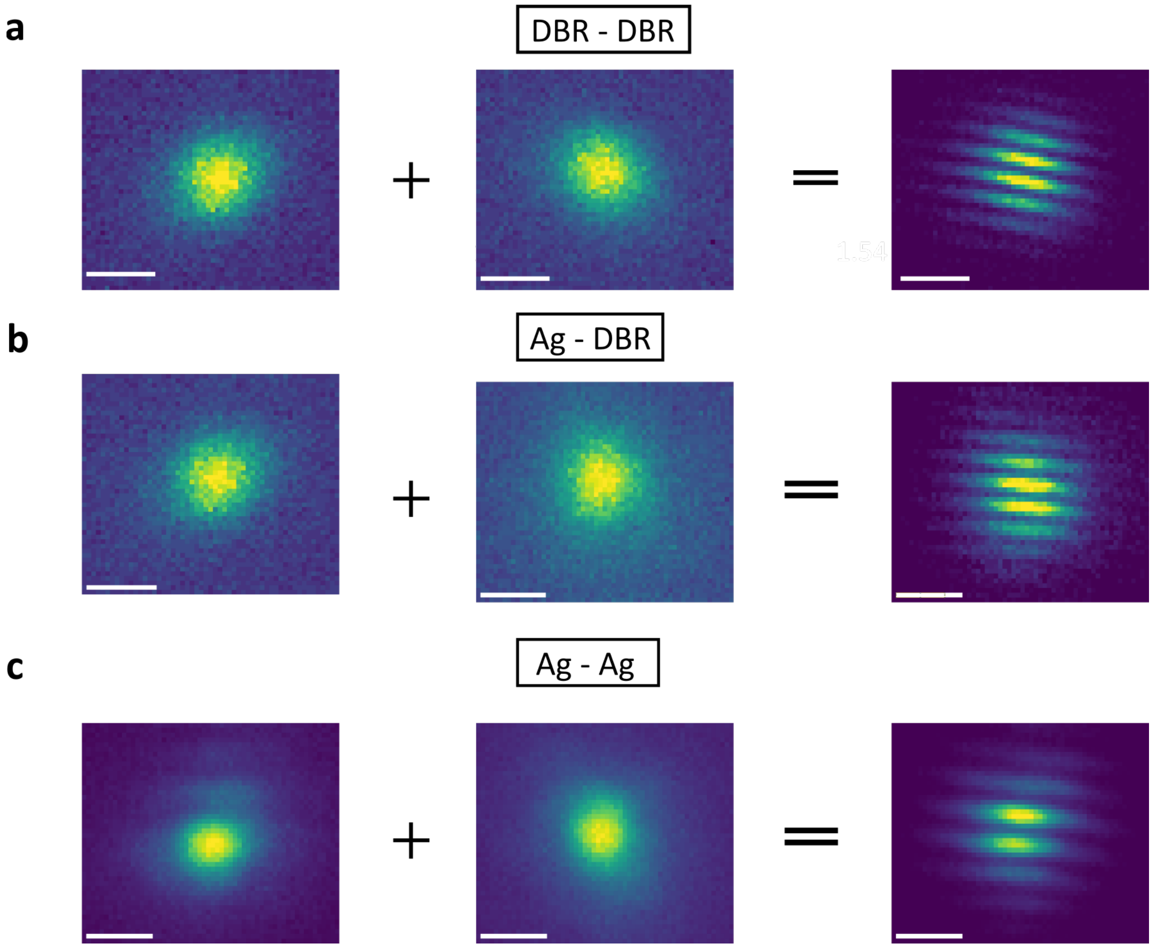

Supplementary Figure 8. Spatial coherence of lasing emission from different cavities. Interferometry measurements performed on a) the DBR/DBR, b) the Ag/DBR, c) and the Ag/Ag cavities using a Michelson interferometer in the retroreflector configuration[6,7]. Scale bars; 2.5 μm.

Supplementary Note 7 - Transfer matrix calculations and Q-factor study

When nominal mirror reflectivity is continuously increased, the Q-factor achievable in optical microcavities made from such mirrors usually saturates at some point due to losses from fabrication limitations. Interestingly, a roughness of around 1 nm is not sufficient for observing the confined emission as seen in Section III an in fact is detrimental to the Q factor. TMCs in **Supplementary Figure 9**a indicated that as little as 1 nm roughness will lead to a 4-fold reduction in the Q-factor of an aligned DBR/DBR cavity. The importance of roughness decreases as the top Ag mirror is added (with a 1 nm roughness accounting for a more modest 21% Q-factor reduction for the Ag/DBR relative to the ideal case), as the linewidth is already broadened by the presence of the lossy metal mirror (Supplementary Figure 9d), suggesting the Ag/DBR is less prone to fabrication imperfections than the DBR/DBR cavity. The increase in Q -factor for the DBR/DBR and Ag/DBR cavities from the ideal values of $Q_{\text{DBR/DBR}}$ = 2661 (Supplementary Figure 9a) and $Q_{\text{Ag/DBR}}$ = 200 (Supplementary Figure 9e) up to the experimental $Q_{\text{DBR/DBR-exp}}$ = 5300 (Supplementary Figure 9c) and $Q_{\text{Ag/DBR-exp}}$ = 333 (Supplementary Figure 9d) can be interpreted as Q-factor enhancement induced by in-plane confinement. Losses increase when the top Ag mirror is evaporated directly on the polymer layer as the metal film formation becomes prone to cracks and imperfections leading to $Q_{\text{Ag-Ag-exp}}$ = 67 (Supplementary Figure 9h), $Q_{\text{Ag-Ag-exp}}$ is however still higher than the Q-factor of an ideal Ag/Ag cavity with first-order design (Supplementary Figure 9i). For these cavities, the ideal Q-factor decreases to 53 and no lasing was observed.

The corresponding TMCs are detailed below. First the results for a DBR/DBR cavity containing a 132 nm-thick aligned layer of PFO with no roughness was simulated. The results are shown in Supplementary Figure 9a. The broadening of the LP is expected to be mainly due to homogeneous broadening[9] with a corresponding Lorentzian width of 1 meV, corresponding to a Q-factor of 2661. Experimental observations[3] show that this value is not reached in an actual cavity, instead, the experimental Q-factor is closer to 500 – 600 and the measured linewidth of the LP is approximately 5 meV.

We suggest that the main source of losses is the roughness of the polymer film as AFM measurements[8] indicate a roughness of at least 1 nm for typical LCCP films. TMCs simulating a variation in roughness were performed taking the average of 10 separate simulations where for i in [1,10] $d(i) = 132 + i \times 0.1$ nm. As i increases, the position of the LP red-shifts with the resulting average reflectivity $R = \sum \frac{R_i}{10}$ shown in Supplementary Figure 8b. The LP mode

was fitted using a Gaussian with linewidth 4.02 meV (Q = 662) demonstrating that roughness can easily account for a 4-fold increase in linewidth and corresponding reduction in Q-factor.

Similar calculations were performed for the Ag/DBR cavity. Supplementary Figure 9d shows the TE-reflectivity spectrum at normal incidence for the direction parallel to the orientation of PFO obtained without any roughness consideration, yielding a fitted linewidth of 13.33 meV (Q ~ 200). Supplementary Figure 9d shows the result for a 1 nm roughness with a linewidth increase to 16.78 meV and a Q-factor reduction down to 158. Compared with the DBR/DBR cavity, the roughness this time only accounts for a 1.25-fold increase in linewidth as the optical losses of Ag now represents the main source of losses.

TMCs were also performed for the Ag/Ag cavity. Supplementary Figure 9g shows the TE-reflectivity spectrum without any roughness consideration; the fitted linewidth is 21.2 meV and Q = 125. The Q-factor is then reduced due to fabrication limitations of the top Ag evaporations.

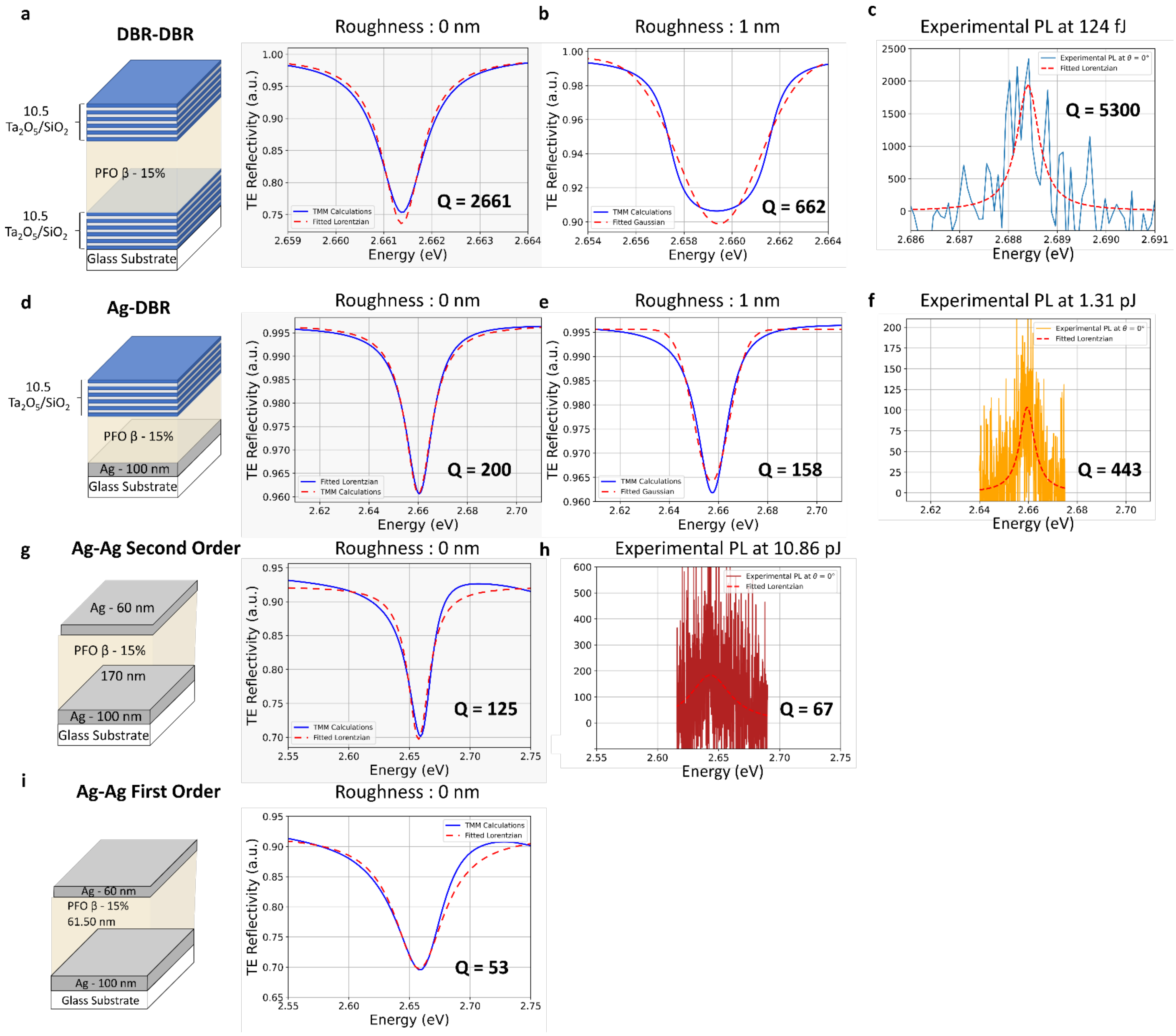

Supplementary Figure 9. *Q*-Factor comparison. Schematic illustrations of the a) DBR/DBR, d) Ag/DBR and g) Ag/Ag second order, and i) Ag/Ag first order cavities used as models in the transfer matrix calculations. Directly to the right of each schematic are the TE-reflectivity spectra obtained from TMC assuming no roughness. b), e) and h) show the corresponding TE-reflectivity spectra after including a 1 nm roughness again obtained via TMC. c), f), h) show the experimentally recorded emission spectra below threshold for θ = 0°.

Supplementary References

1. https://www.lumerical.com/ansys/
2. Ohzono, T., Katoh, K.,Wang, C., Fukazawa, A., Yamaguchi, S., Fukuda, J-I. Uncovering different states of topological defects in schlieren textures of a nematic liquid crystal. *Scientific Reports* **7**, 16814 (2017).
3. F. Le Roux, A. Mischok, D. D. C. Bradley & M. C. Gather. Efficient anisotropic polariton lasing using molecular conformation and orientation in organic microcavities. *Adv Functional Materials* **32**, 2209241(2022).
4. Ishii, T. *et al.* Low-Threshold Exciton-Polariton Condensation via Fast Polariton Relaxation in Organic Microcavities. Adv Opt Mater **10**, (2021).
5. Ishii, T. *et al.* Enhanced Light–Matter Interaction and Polariton Relaxation by the Control of Molecular Orientation. *Advanced Optical Materials* **9**, (2021).
6. Daskalakis, K. S., Maier, S. A., Murray, R. & Kéna-Cohen, S. Nonlinear interactions in an organic polariton condensate. *Nature Materials* **13**, 271–278 (2014).
7. Plumhof, J. D., Stöferle, T., Mai, L., Scherf, U. & Mahrt, R. F. Room-temperature Bose-Einstein condensation of cavity exciton-polaritons in a polymer. *Nature Materials* **13**, 247–252 (2014).
8. Zhang, H. *et al.* Azobenzene Sulphonic Dye Photoalignment as a Means to Fabricate Liquid Crystalline Conjugated Polymer Chain-Orientation-Based Optical Structures. *Advanced Optical Materials,* **8***,* 1901958 (2020).
9. Houdré, R., Stanley, R. P. & Ilegems, M. Vacuum-field Rabi splitting in the presence of inhomogeneous broadening: Resolution of a homogeneous linewidth in an inhomogeneously broadened system. *Physical Review A* **53** 2711 (1996).
