# Supplementary material for: Schlieren texture and topography induced confinement in an organic exciton-polariton laser
Source: Nat Commun. 2025 Jan 18;16:811. doi: 10.1038/s41467-025-55875-1 (PMC11743153; doi:10.1038/s41467-025-55875-1)
Supplement: Supplementary file 2 — Transparent Peer Review file [file 41467_2025_55875_MOESM2_ESM.pdf]

nature portfolio

Peer Review File

Schlieren texture and topography induced confinement in an organic exciton-polariton laser

Corresponding Author: Professor Malte Gather

This manuscript has been previously reviewed at another journal. This document only contains information relating to versions considered at Nature Communications.

Version 0:

Reviewer comments:

Reviewer #1

(Remarks to the Author)
I thank the authors for their additional comments and modifications. I recommend the revised version of the manuscript for publication in Nature Communications. I find the novelty and quality of the work well suited for this journal.

Open Access This Peer Review File is licensed under a Creative Commons Attribution 4.0 International License, which permits use, sharing, adaptation, distribution and reproduction in any medium or format, as long as you give appropriate credit to the original author(s) and the source, provide a link to the Creative Commons license, and indicate if changes were made.
In cases where reviewers are anonymous, credit should be given to 'Anonymous Referee' and the source.
The images or other third party material in this Peer Review File are included in the article's Creative Commons license, unless indicated otherwise in a credit line to the material. If material is not included in the article's Creative Commons license and your intended use is not permitted by statutory regulation or exceeds the permitted use, you will need to obtain permission directly from the copyright holder.
To view a copy of this license, visit https://creativecommons.org/licenses/by/4.0/
